# Insights into Within-Host Evolution and Dynamics of Oral and Intestinal Streptococci Unveil Niche Adaptation

**DOI:** 10.3390/ijms252413507

**Published:** 2024-12-17

**Authors:** Mohamed M. H. Abdelbary, Maximilian Hatting, Andrea Dahlhausen, Alexandra Bott, Georg Conrads

**Affiliations:** 1 Division of Nosocomial Pathogens and Antibiotic Resistances, Department of Infectious Diseases, Robert Koch Institute, Wernigerode Branch, 38855 Wernigerode, Germany; 2 Division of Oral Microbiology and Immunology, Department of Operative Dentistry, Periodontology and Preventive Dentistry, Rheinisch-Westfälische Technische Hochschule (RWTH) University Hospital, 52074 Aachen, Germany; alexandra.bott@rwth-aachen.de (A.B.); gconrads@ukaachen.de (G.C.); 3 Department of Medicine III, RWTH University Hospital, 52074 Aachen, Germany; max.hatting@live.com; 4 University Medical Center for Occupational Medicine, RWTH University, 52074 Aachen, Germany; dahlhausen@hsa.rwth-aachen.de

**Keywords:** within-host evolution, streptococci, oral–gut axis, inflammatory bowel disease, mobile genetic elements, recombination, mutational spectrum

## Abstract

The oral–gut axis is a complex system linking the oral cavity and gastrointestinal tract, impacting host health and microbial composition. This study investigates genetic changes and adaptive mechanisms employed by streptococci—one of the few genera capable of colonizing oral and intestinal niches—within the same individual. We conducted whole-genome sequencing (WGS) on 218 streptococcal isolates from saliva and fecal samples of 14 inflammatory bowel disease (IBD) patients and 12 healthy controls. Our analysis identified 16 streptococcal species, with *Streptococcus infantis*, *S. mitis*, *S. parasanguinis*, *S. australis*, and *S. salivarius* being the most prevalent. *S. infantis* dominated the oral niche in both IBD patients (33%) and healthy controls (26%). It was also the primary species in fecal samples from IBD patients and the second most prevalent in those from healthy controls. *S. parasanguinis* was more prevalent in the gut than in the oral cavity in both groups. Comparative genomics demonstrated a within-host microevolution of streptococci, showing adaptations via recombination and acquisition of mobile genetic elements (MGEs). Intestinal streptococcal genomes exhibited a higher proportion of intact phages and a significantly greater acquisition of the *tetA* gene, which confers tetracycline resistance compared to oral genomes. Core-genome single-nucleotide polymorphisms (SNPs) analysis showed significant genetic divergence between oral and intestinal streptococcal genomes within the same individual. Our findings also unveil distinct niche-specific mutation signatures within intestinal genomes, indicating the emergence of distinct clonal lineages within each niche and suggesting that within-host streptococcal evolution is individual-dependent, initiated in the oral cavity.

## 1. Introduction

The human microbiome stands as a complex ecosystem, harboring a myriad of microbial communities that orchestrate indispensable roles in host health and disease. Among these communities, oral and intestinal streptococci emerge as pivotal constituents, exerting substantial influence over host physiology and immune responses. Streptococci, characterized by their diversity, are renowned for their capacity to instigate a broad spectrum of infections in humans. These bacteria are ubiquitous across anatomical sites including the oral cavity, respiratory tract, skin, and gastrointestinal system, contributing to diseases spanning from benign infections like strep throat to severe conditions such as pneumonia, endocarditis, and necrotizing fasciitis [1]. Notable members of this bacterial group, such as *Streptococcus mutans* and *S. salivarius*, hold prominence within the oral microbiota, whereas others like *S. anginosus* and *S. thermophilus*, inhabit the gastrointestinal tract. Through multifarious mechanisms encompassing nutrient metabolism, mucosal barrier maintenance, and immunomodulation, streptococci intricately contribute to host physiology. However, oral and intestinal streptococci are also implicated in various diseases and conditions including dental caries, periodontal disease, gastrointestinal infections, and systemic diseases such as endocarditis [2].

The oral cavity and intestine are intricately interconnected through the oral–gut axis, facilitating bidirectional communication between these disparate microbial ecosystems. Dysregulation of this axis has been implicated in a spectrum of systemic diseases including cardiovascular diseases, metabolic disorders, autoimmune conditions, and inflammatory bowel diseases (IBD) [3,4]. Within-host evolution delineates the genetic changes occurring within microbial populations during infection within a single host [5]. Within the human body, streptococci encounter a dynamic and challenging environment brimming with myriad opportunities for evolutionary adaptation and diversification. Possessing substantial genetic plasticity akin to other bacterial pathogens, streptococci competently adapt to selective pressures imposed by the host immune system, antimicrobial therapies, and local microenvironmental shifts. While previous studies have predominantly focused on the within-host evolution of pathogenic streptococcal species such as *S. pneumoniae* during infection, they have unveiled marked genetic diversity within streptococcal populations, driven by mechanisms including mutation, recombination, and horizontal gene transfer [6]. These studies have illuminated the pivotal role of factors such as the high replication rate of bacteria, genetic variability, selective pressures from the host immune system, and antimicrobial interventions in shaping streptococcal evolution. These factors foster the accumulation of genetic mutations, gene transfer events, and selection of adaptive traits within the streptococcal population, culminating in the emergence of strains with altered phenotypic characteristics. The genetic diversity that streptococci exhibit appears to give them the adaptability they need to thrive in different environmental niches within the host milieu, which include both the oral cavity and the gastrointestinal tract. Despite burgeoning research endeavors into streptococcal biology, substantial lacunae persist in our comprehension of the within-host evolution and dynamics of oral and intestinal streptococci. Consequently, unraveling the within-host evolution and adaptation of these streptococci assumes paramount importance for elucidating their roles in disease pathogenesis, transmission dynamics, and the genesis of antimicrobial resistance [7,8]. Previous investigations carried out by our research team have revealed the existence of several clusters specific to patients with IBD, comprising streptococcal isolates obtained from both saliva and feces of the same individual and belonging to the same species. These findings established a correlation between streptococcal strains found in the oral and gut environments of patients with IBD, indicating that the oral cavity may indeed act as an intrinsic reservoir for intestinal strains [3]. However, the precise mechanisms and dynamics governing within-host evolution in these niches remain incompletely understood. Further investigations are warranted to unravel the intricacies of within-host streptococcal evolution, shedding light on its population dynamics and the development of adaptive traits to different niches. Such insights hold promise for informing therapeutic strategies aimed at mitigating the impact of streptococcal infections and restoring host-microbiome homeostasis. Hence, this study aims to scrutinize the population dynamics, genetic alterations, and adaptive strategies employed by streptococci during colonization of both oral and intestinal niches within patients afflicted by IBD and healthy controls, leveraging whole-genome sequencing methodologies. Through comprehensive genomic analyses, this investigation seeks to shed light on the intricate interplay between streptococci and their host environments, unraveling novel insights into their adaptive mechanisms and within-host evolution.

## 2. Results

### 2.1. Oral and Intestinal Colonization Dynamics of Streptococci

In this study, the WGS of 218 streptococcal isolates was performed and analyzed. Among them, 93 and 55 isolates were retrieved from saliva samples of IBD patients and healthy control subjects, respectively. Additionally, 33 isolates were retrieved from fecal samples of IBD patients, while 37 isolates were obtained from fecal samples of healthy control subjects (Table S1). The mean number of salivary streptococcal isolates retrieved per IBD patient was 6.6 (range: 2–14), while the mean per healthy control was 5 (range: 1–9). For the fecal streptococcal isolates, a mean of 2.4 (range: 0–8) and 3.4 (range: 2–5) was detected per IBD patient and healthy control, respectively. On the other hand, the mean number of colonizing salivary streptococcal species per IBD patient was 4.1 (range: 2–8), while the mean per healthy control was 3.9 (range: 1–7). For the streptococcal species colonizing the gut, a mean of 2 (range: 0–5) and 3 (range: 1–5) was detected per IBD patient and healthy control, respectively.

We analyzed the genome sequences of 218 streptococcal isolates, and *in silico* species identification revealed the presence of 16 distinct *Streptococcus* species. Among these, *S. infantis*, *S. mitis*, *S. parasanguinis*, *S. australis*, and *S. salivarius* were the most predominant species (Figure S1). Notably, *S. infantis* was the most prevalent species identified in the oral niche of both IBD patients and healthy control subjects, comprising 33% and 26% of the total genomes analyzed, respectively. Furthermore, *S. infantis* was also the predominant species in fecal samples from IBD patients and the second most prevalent in those from healthy controls.

**Figure 1:**
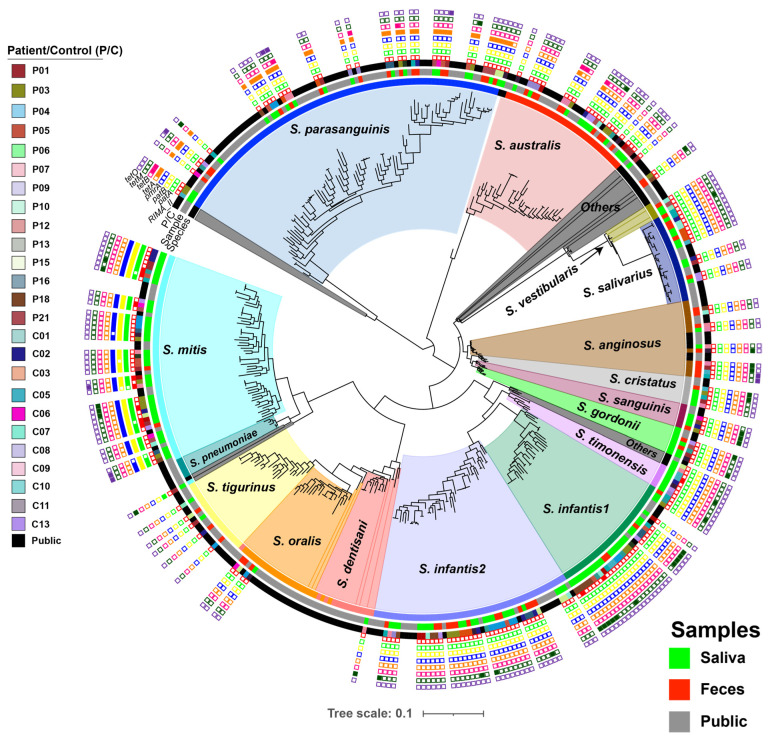
Dynamics of streptococcal colonization in oral and intestinal niches. A mid-rooted maximum-likelihood (ML) phylogenetic tree was constructed using 218 streptococcal genomes, based on pan-core-genome SNPs. Clade colors indicate different streptococcal species. The tree is annotated with rings, each conveying distinct information. The innermost ring (Species) represents the detected species among the 218 genomes. The second ring (Sample) categorizes these genomes based on their sample origin, with saliva samples shown in green, fecal samples in red, and publicly retrieved genomes in gray. The third ring (Pt./Cont.) categorizes samples based on their origin from each IBD patient (Pt.) or healthy control (Cont.) individual. Subsequent rings (fourth to eleventh) represent the presence or absence of the following resistance genes: *rlmA(II)*, *patA*, *patB*, *pmrA*, *tetA*, *tetB*, *tetM*, and *tetO*, respectively. Each gene’s presence is denoted by a filled square, while absence is indicated by an unfilled square.

A pan-genome analysis was performed on the entire set of 218 streptococcal genomes, representing multiple species. This analysis identified pan-core-genome SNPs across a core-genome alignment of 12,369 base pairs (bp). These SNPs were subsequently used to reconstruct the phylogeny of the isolates using a maximum-likelihood (ML) approach (Figure 1). The resulting phylogenetic tree delineated the isolates into 16 distinct species-specific clades, each labeled according to the corresponding species. For instance, all genomes classified as *S. parasanguinis* formed a distinct clade labeled *S. parasanguinis*. Similarly, genomes of *S. mitis*, *S. australis*, *S. salivarius*, and *S. anginosus* each clustered into their respective species-specific clades (Figure 1). Interestingly, the phylogenetic analysis revealed that *S. infantis* genomes were divided into two phylogenetically distinct clades, designated *S. infantis1* and *S. infantis2* (Figure 1). Notably, *S. infantis1* genomes were predominantly identified in the oral cavity, while *S. infantis2* genomes were more frequently observed in the gut of IBD patients (n = 8) compared to *S. infantis1* (n = 1). In addition to *S. infantis*, *S. mitis* emerged as the second most dominant species among salivary streptococcal isolates, constituting 30% of the isolates in IBD patients and 20% in healthy controls. However, it made up only 3% of the fecal isolates in each of IBD and controls. In general, *S. parasanguinis* was more abundant in the gut of both healthy controls (16%) and IBD patients (30%) than in their oral niches (7% and 17%, respectively). Interestingly, IBD patients harbored more *S. parasanguinis* than healthy controls (Figure S1A). In contrast, isolates belonging to *S. australis* were more abundant in the oral and gut niches of healthy controls (11% and 16%, respectively) than in their IBD counterparts (6% and 9%, respectively). Similarly, *S. salivarius* was mainly detected in the oral and gut niches of healthy controls (13% and 14%, respectively) and they only made up 1% of the salivary streptococci isolated from IBD patients (Figure S1A).

All 218 streptococcal genomes underwent comprehensive *in silico* screening to identify antimicrobial resistance genes, meticulously documented in the Supplementary Table S2. Notably, our analysis revealed a significant correlation between the oral origin and four specific antimicrobial resistance genes: *rlmA(II)* (*p* = 0.018), *patA* (*p* = 0.0004), *patB* (*p* = 0.0002), and *pmrA* (*p* = 0.0002) (Figure 1 and Figure S1B).

The *rlmA(II)* gene, a recognized housekeeping methyltransferase previously identified in *S. pneumoniae*, exhibited a prominent presence and was associated with resistance to tylosin and mycinamicin when combined with the ErmN monomethylase [9]. Similarly, the presence of *patA*, *patB*, and *pmrA* genes correlated with fluoroquinolone resistance in *S. pneumoniae* [10]. Additionally, it is noteworthy that the *tetA* gene, responsible for conferring resistance to tetracycline, was significantly (*p* = 0.04) more abundant in genomes originating from fecal sources (Figure 1 and Figure S1B).

### 2.2. Within-Host Evolution of Streptococci and Their Adaptation to the Oral-Gut Niches

To explore the within-host evolution of streptococcal strains, we introduced the term “oral–gut pair,” denoting the identification of isolates from the same species at one or more sequential sampling points in both salivary and fecal samples of an individual, whether they were IBD patients or were healthy control subjects. Our definition unveiled 30 oral–gut pairs of streptococcal isolates, recovered from 9 out of 11 healthy controls and 11 out of 14 IBD patients, exhibiting mean pairs/group of 1.6 (range: 1–3) and 1.5 (range: 1–3), respectively. Predominant species included *S. parasanguinis*, *S. infantis*, *S. salivarius*, *S. australis*, and *S. mitis*, comprising 12, 7, 5, 5, and 1 pairs of oral–gut isolates, respectively.

Subsequently, we examined the contribution of mobile genetic elements (MGEs) and non-MGE genes to the diversity of oral and intestinal genomes. Our comparative genomic analysis uncovered distinct within-host evolutionary trajectories for oral and intestinal streptococcal genomes. For instance, the oral and intestinal genomes of *S. parasanguinis* from P07 and P016 displayed striking similarity and minimal unique gene acquisition (Table 1). Similarly, genomes from P04 and P15 exhibited limited diversity, notably in phage acquisition and vitamin B12 synthesis gene clusters (Table 1 and Supplementary File S1). In contrast, hosts such as C03, C06, P05, P03, or P09 manifested substantial unique gene acquisition in their oral and intestinal *S. parasanguinis* genomes, primarily attributed to genetic content such as phages, lactose transport systems, type I restriction systems, vitamin B12 synthesis, and various gene clusters implicated in DNA damage repair mechanisms associated with antibiotics and immune responses (e.g., *xerC* and *dnaG*; see Supplementary File S1). Furthermore, we analyzed four *S. parasanguinis* genomes from P21 (2 oral and 2 intestinal), revealing considerable variability in MGEs, including the accessory secretion system and ABC transporter, even within the same niche (Supplementary File S1). Similar to *S. parasanguinis*, distinct evolutionary patterns were observed in the genomes of *S. infantis* (*S. infantis1* and *S. infantis2*), *S. salivarius*, and *S. australis*. For instance, oral and intestinal genomes belonging to *S. infantis2* from P04 and P16 exhibited minimal variation in MGEs, except for C03, whose intestinal genome harbored Type III restriction-modification system genes (Supplementary File S1).

Conversely, in hosts like P15, P18, and C02, three oral and intestinal *S. infantis2* genomes were analyzed per individual. It is noteworthy that P18’s intestinal genome OMI885 exhibited a significant divergence (*p* < 0.0001) from OMI702 (oral) due to differences in genetic elements such as phage content and fused mannose-specific PTS enzymes. In contrast, C02’s two intestinal genomes (OMI816 and OMI802) exhibited substantial dissimilarity, with OMI802 resembling the oral genome OM813, albeit with variations including *tetM* and fused mannose-specific PTS enzymes (Supplementary File S1). However, no significant difference in genome length was detected among oral and intestinal genomes (Table S1). Taken together, these findings suggest the emergence of distinct clonal lineages within each niche and indicate that the within-host evolution of streptococci likely initiates in the oral cavity. Furthermore, MGEs, such as phages, exert a crucial influence on the evolution and adaptation of streptococcal strains to the intestinal niche by promoting horizontal gene transfer, thereby facilitating the acquisition of novel genetic traits, including antibiotic resistance genes.

### 2.3. Streptococcal Phage Dynamics Across the Oral–Gut Axis

Analysis of the phage gene content within 218 genomes spanning various species of oral and intestinal origin revealed that intestinal streptococcal genomes exhibited a slightly higher proportion of intact phages (detection score > 90) [11,12] and questionable phages (detection score 70–90) compared to their oral counterparts (11.25% vs. 8.3% and 16.25% vs. 13.9%, respectively). However, the percentage of incomplete phages (detection score < 70) detected in intestinal genomes was marginally lower (73.13%) than in oral genomes (77.8%). Further examination at the species level unveiled a notable discrepancy in the average phage content among different streptococcal species. Among all species analyzed, *S. parasanguinis* exhibited the highest proportion of intact phages in both oral and intestinal niches (15.8% and 16.7%, respectively), followed by *S. infantis* and *S. australis*. Conversely, oral genomes of *S. parasanguinis* displayed the lowest proportion of questionable phages (2.6%), followed by their intestinal counterparts (5.6%). An intriguing observation emerged regarding the *S. infantis1* genomes, which showed the highest content of questionable phages among all species (30.4%), along with a reduction in incomplete phage content to 63.8%. This marked the lowest value among incomplete phages compared to other species, ranging from 76.7% in intestinal genomes of *S. infantis2* to 88.9% in intestinal genomes of *S. australis*.

**Figure 2:**
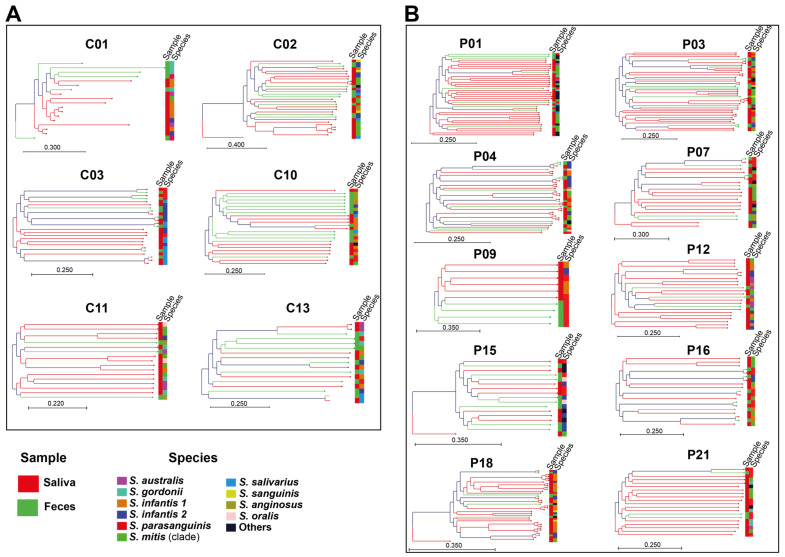
Cross-species and cross-niche transmission of streptococcal phages. Phylogenetic *k*-mer-based trees of phage sequences from oral and intestinal niches of healthy controls (**A**) and IBD patients (**B**), disregarding species delineation. Each phylogenetic tree represents all phages found in each individual (“C” stands for control, and “P” for patient). The branches and leaves are colored according to the different niches: red for oral and green for intestinal. On the right-hand side, the first lane indicates the sample of origin (saliva: red; feces: green), while the second lane represents the different streptococcal species. Evidence for cross-species transmission of phages can be seen in cases such as C10 (in saliva and fecal samples) and P12 (in saliva samples), while evidence for cross-niche transmission of phages is represented in cases such as C03 (by *S. parasanguinis* and *S. salivarius)* and P09 (by *S. parasanguinis*).

To elucidate the diversity, abundance, and spatial distribution of phage populations within the host, we conducted a comprehensive phylogenetic analysis encompassing all phage sequences identified within both the oral and intestinal niches of individual hosts (IBD and healthy controls) without regard to species delineation. Our analysis unveiled a spectrum of major and minor clusters delineating oral and intestinal phages, exhibiting distinct patterns reflective of niche specificity (Figure 2). For instance, individuals C01, C10, C13, P01, P09, P12, and P21 manifested discernible major clusters predominantly comprising either oral or intestinal phages. Conversely, individuals such as C02, C13, P03, P04, and P15 exhibited minor clusters primarily associated with phages originating from specific niches (Figure 2). Notably, our investigation revealed instances where phages derived from different streptococcal species within the same host displayed phylogenetic clustering similarities. Furthermore, oral and intestinal phages from disparate species formed cohesive clusters, as observed in cases such as C02, C11, P15, and P16 (Figure 2). Of particular interest was the observation that despite the genetic relatedness of their phages, streptococcal species did not consistently align phylogenetically. This was evidenced by the case of *S. australis* (mitis group) and *S. gordonii* (sanguinis group) within individual C11. These findings emphasize the prevalence of cross-niche and cross-species transmissions among streptococcal phages, highlighting a previously underappreciated aspect of their microbial ecology.

### 2.4. Emergence of Diverse Streptococcal Clonal Lineages Within-Hosts

The genetic diversity and clonal relationships within the host were assessed for each of the four streptococcal species mentioned (including both *S. infantis1* and *S. infantis2*) by aligning their genomes to publicly available reference genomes and calculating the number of pairwise core-genome SNP differences (PWSD), as outlined in the methods section.

**Figure 3:**
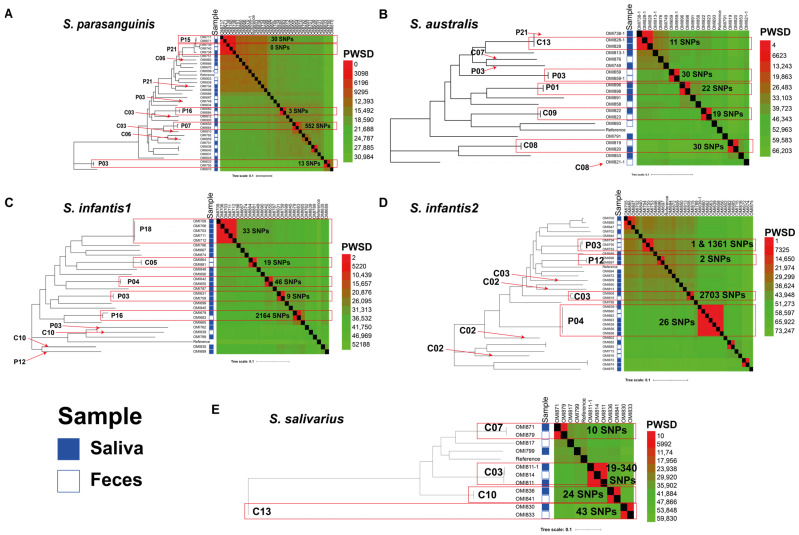
Within-host streptococcal genetic diversity. Mid-rooted phylogenetic trees based on core-genome SNPs of the four main streptococcal species investigated in this study: *Streptococcus parasanguinis* (**A**), *S. australis* (**B**), *S. infantis*, including both *S. infantis1* (**C**) and *S. infantis2* (**D**), and *S. salivarius* (**E**). The heat maps on the right side represent the pairwise core-genome SNP differences (PWSD) that were calculated by aligning their genomes to publicly available reference genomes as outlined in the method section. The mean genetic diversity varied between pairs of strains and niches for the same species within the same individual on multiple occasions, as indicated by the red arrows and boxes.

Notably, the mean genetic diversity varied between pairs of strains and niches for the same species within the same individual on multiple occasions (Table S3). For instance, the PWSD between oral and intestinal *S. parasanguinis* genomes of individuals P07, P15, and P16 were 552, 30, and 3 PWSDs, respectively. Conversely, for individuals, C08 and C09, the oral and intestinal pairs of *S. australis* genomes exhibited 30 and 19 PWSDs, respectively (Figure 3). Similarly, genomes from the same niche within the same individual displayed a low number of PWSDs; for example, the oral *S. parasanguinis* and *S. infantis1* genomes of individual P03 had 13 and 9 PWSDs. Comparable findings were observed for the oral *S. australis* genomes of individuals C13 and P01, with 11 and 22 PWSDs, respectively, as well as for the oral *S. infantis1* genomes of individuals P18 (n = 5) and P04, which had 33 and 46 PWSDs, respectively. However, some individuals exhibited an unusually high number of PWSDs between specific genomes, even within the same niche (Figure 3). For instance, the oral and intestinal genomes of *S. infantis2* from individual C03 and intestinal genomes of individual P03, had 2703 and 1361 PWSDs, respectively. Additionally, it is noteworthy that four pairs of oral and intestinal genomes of *S. salivarius* were identified among healthy controls (individuals C07, C10, C13, and C03, with three genomes). These pairs were highly conserved, with PWSDs ranging from 10 to 340 SNPs (Figure 3). These findings collectively suggest an interplay between host and bacterial factors influencing within-host streptococcal genetic diversity. Furthermore, it can be hypothesized that oral and intestinal divergent strains may have originated from ancestral strains during colonization via within-host homologous recombination, leading to rapid accumulation of genetic variation.

**Figure 4:**
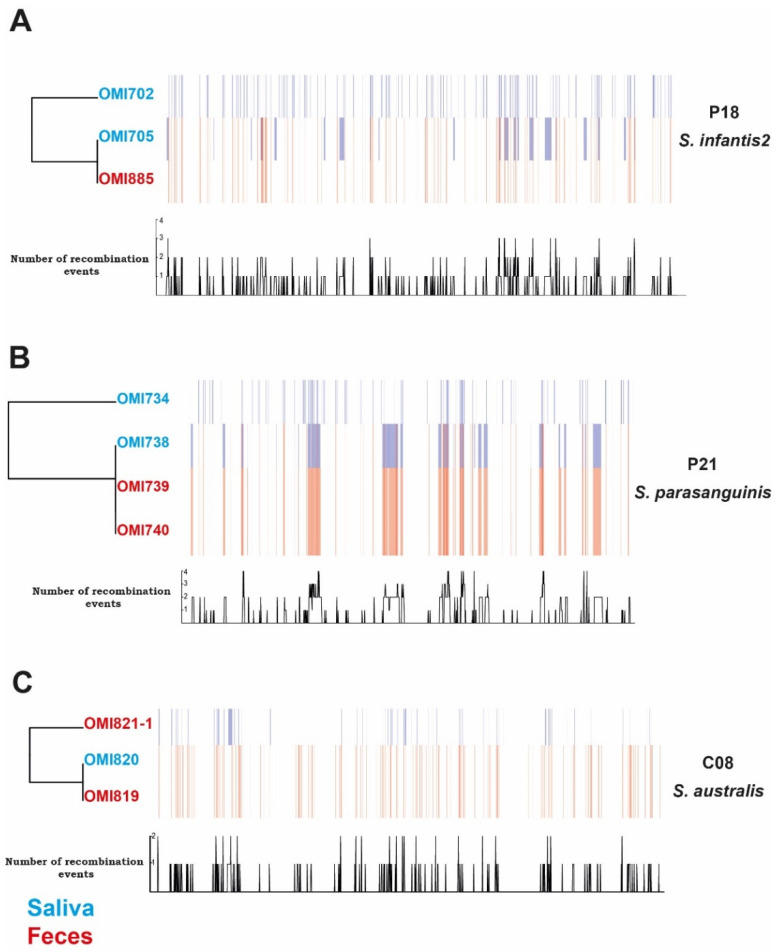
Within-host streptococcal recombination. Gubbins-based recombinational analysis and phylogenetic tree reconstruction illustrate oral and intestinal streptococcal genomes from the same host, revealing a high frequency of recombination events. Red blocks show predicted recombination on internal branches, indicating shared ancestry among multiple isolates. Conversely, blue blocks denote recombination events on terminal branches, highlighting their uniqueness within individual isolates. The black bars represent the number of observed recombination events covering specific positions in the genome.

To test this hypothesis, we characterized the signatures of within-host streptococcal recombination for all individuals possessing at least three genomes of the same species, as described in the methodology. Our analysis revealed individual-dependent differences in recombination rates between species (Figure 4 and Figure S2). For instance, the *S. infantis2* genomes from individuals C02 and C03 exhibited few recombination events (n = 28 and n = 74, respectively) (Figure S2). In contrast, a high rate of recombination events was observed in genomes from the same *S. infantis2* clade in P18, *S. parasanguinis* P21, and *S. australis* C08, with 297, 323, and 342 recombination events, respectively (Figure 4). Conversely, certain individuals, such as individual P03 (with *S. parasanguinis* and *S. infantis1* genomes), exhibited a notably high level of divergence among their genomes (>25%), posing challenges to homologous recombination analysis using Gibbins. Additionally, we observed distinct recombination patterns in oral genomes, with unique events in individuals P18 (OMI702) and P21 (OMI734), clustering as an outgroup (depicted as blue blocks in Figure 4). Conversely, other oral genomes from the same hosts, such as those in individuals P18 (OMI705) and P21 (OMI738), exhibited their unique recombination events but clustered with intestinal genomes from the same hosts, namely P18 (OMI885) and P21 (OMI739 and OMI740), and shared recombination events with these intestinal genomes (indicated by red blocks in Figure 4). These results underscore the genome plasticity of streptococci facilitated by within-host homologous recombination, emphasizing that the common ancestor of all genomes of individuals P18 and P21 originated from the oral niche.

### 2.5. Mutational Spectra Shaped by Oral and Intestinal Niches

Utilizing the MutTui pipeline, we conducted a meticulous reconstruction of mutational spectra resulting from single base substitutions (SBS) within the principal streptococcal species (*S. salivarius*, *S. infantis*, *S. parasanguinis*, and *S. australis*). This mutational spectrum involves a detailed analysis of the specific patterns and frequencies of nucleotide changes (e.g., A→T, G→C) across a genome. It provides a fingerprint of the mutational processes influencing the four streptococcal species’ genetic makeup. These species were prevalent in both oral and intestinal niches, encompassing the two distinct phylogenetic clades of *S. infantis* (*S. infantis1* and *S. infantis2*). Our investigation unveiled diversity within the SBS spectra, capturing variations not only in the nucleotide mutations themselves but also in their surrounding contexts (Figure S3). Our comprehensive analysis demonstrated that, on average, transition mutations account for a higher percentage (73.9% in oral and 72% in intestinal) compared to transversion mutations (26.1% in oral and 28% in intestinal) across all four investigated streptococcal species. Furthermore, our results revealed that mutations involving cytosine and thymine (C>T and T>C) are consistently the most prevalent, regardless of the species or niche of origin for the genomes (Figure S3).

**Figure 5:**
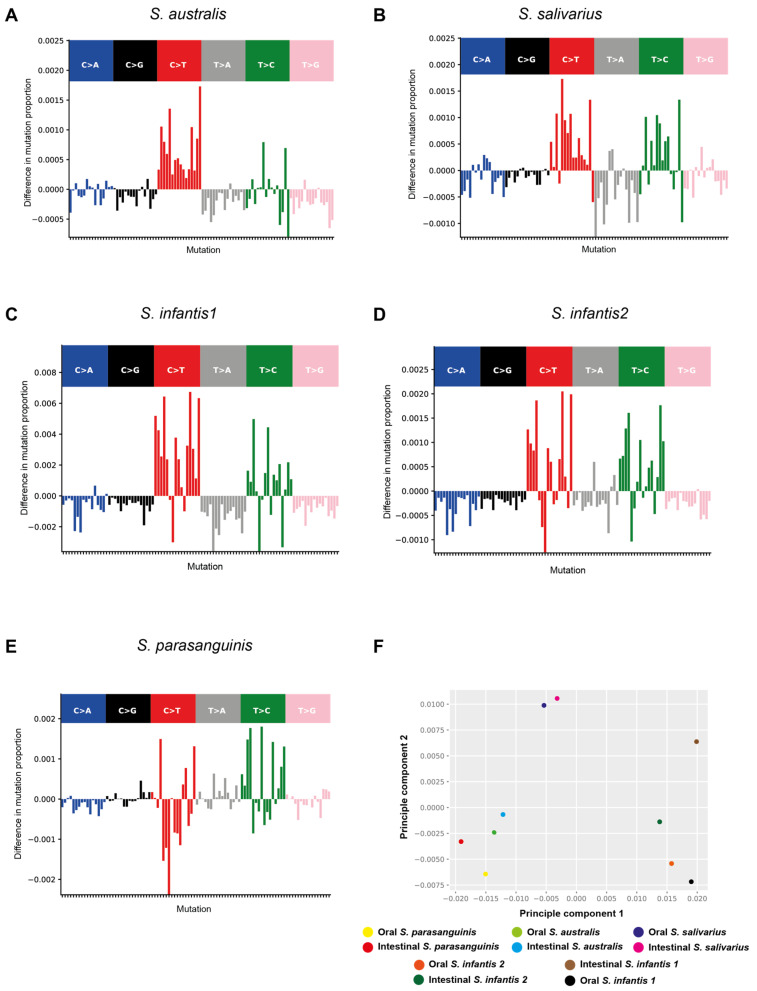
Comprehensive comparison of mutational spectra between the oral and intestinal niches of the four investigated streptococcal species. These spectrum plots visually depict variations in mutation proportions between the oral and intestinal niches, as quantified by the MutTui pipeline. Panels (**A**–**E**) showcase spectrum plots representing the difference in mutation proportions for *Streptococcus australis*, *S. salivarius*, *S. infantis1*, *S. infantis2*, and *S. parasanguinis*, respectively. In each panel, the single base substitution (SBS) spectrum of intestinal genomes is subtracted from that of the oral genomes for the same species, highlighting niche-specific mutational differences. In Panel (**F**), a principal component analysis (PCA) is conducted on mutation proportions within the SBS spectra across all four investigated streptococcal species and both oral and intestinal niches. The axes labels provide insights into the inferred proportion of variance described by each principal component. Points on the plot are color-coded based on the niche and species for easy interpretation of the data.

We further aimed to delve into niche-specific mutational signatures by comparing the oral and intestinal spectra for each species (Figure 5). Interestingly, our data unveiled distinctive niche-specific mutation signatures in the intestinal genomes, characterized by a lower proportion of transversion mutations (C>A, C>G, T>A, and T>G), especially notable in *S. australis*, *S. salivarius*, *S. infantis1*, and *S. infantis2*. This lower transversion mutation proportion correlated with an increase in the proportion of C>T and T>C mutations. In contrast, *S. parasanguinis* exhibited different niche-specific mutational signatures, particularly for C>T, which had a lower mutation proportion in intestinal niche genomes (Figure 5). This was coupled with a higher mutation proportion of T>A and a similar mutation proportion of C>G. Moreover, we performed a comparative analysis of the SBS spectra across all four species and for both oral and intestinal niches through principal component analysis (PCA). The results confirmed that each species clusters separately based on the SBS spectrum composition of their niche of origin (Figure 5). Notably, *S. infantis1* and *S. infantis2* displayed very distinct SBS spectrum compositions for their oral and intestinal niches, placing them far apart on the clustering PCA plot. Taken together, these findings suggest that the oral and intestinal niches play a significant role in shaping the mutational spectra of streptococcal species, and these niche-specific mutational signatures are species-dependent.

## 3. Discussion

The findings of our study indicate that *S. infantis* was the predominant species in the oral niche in both IBD patients and healthy controls. Conversely, *S. parasanguinis* exhibited a higher abundance in the gut than in the oral cavity. This predominance may be attributed to the ability of each bacterium to adapt to the distinct physicochemical conditions of the oral cavity and intestine, including variations in pH, oxygen availability, and nutrient sources. For example, *S. infantis* exhibits remarkable metabolic flexibility, enabling it to utilize a diverse range of carbohydrates, including sucrose and other sugars commonly present in the human diet. This adaptability enables the bacterium to outcompete other microbes for essential nutrients, thereby contributing to its ecological success in the oral niche [13]. Conversely, the intestinal environment provides a nutrient-rich and relatively stable habitat, which allows *S. parasanguinis* to flourish and maintain its ecological dominance. The coevolution of *S. parasanguinis* with the host and other gut microbiota has equipped it with effective strategies for resource utilization and niche partitioning within the intestinal ecosystem. For example, prior studies have demonstrated that *S. salivarius* F286 and *S. parasanguinis* F278 may exert anti-inflammatory effects on gut immunity, highlighting their potential as probiotics [14].

Our investigation elucidates a diverse spectrum of genetic alterations occurring during the colonization process of streptococcal species, encompassing the acquisition of MGEs and mutations within the core genome. Notably, we observed the independent emergence of several analogous genetic signatures across different strains of the same streptococcal species, even when colonizing disparate individuals. Moreover, upon expanding the scope of our analysis beyond the comparison of IBD subjects to healthy controls, we identified multiple genetic signatures consistently influencing numerous strains of unrelated streptococcal species, irrespective of the host’s health status. These findings robustly indicate the frequent coexistence of specific genetic signatures, suggesting their potential for conferring advantageous traits within-host environments. Consequently, our observations imply a discernible pattern of adaptive evolution across streptococcal populations, tailored to both oral and intestinal niches.

We observed a significant correlation between the oral niche and the presence of specific antimicrobial resistance genes—*rlmA(II)*, *patA*, *patB*, and *pmrA*—while the *tetA* gene showed a notable association with the intestinal niche. Remarkably, these findings are consistent with a prior investigation comparing the resistomes of the oral and gut microbiota in healthy individuals across diverse geographical regions using shotgun metagenomics [15]. This study revealed a higher relative abundance of antimicrobial resistance genes encoding fluoroquinolone efflux pumps, exemplified by *patB*, in oral samples. Conversely, stool samples exhibited a greater proportion of tetracycline resistance genes. These observations suggest distinct body site-specific differences in the prevalence of antimicrobial resistance genes, classes, and mechanisms between oral and stool samples [15]. However, this study did not delineate a correlation between such site-specific resistance patterns and specific bacterial species. Our results suggest that this association was attributed to the elevated abundance of *S. mitis* and *S. parasanguinis* in the oral and intestinal niches, respectively (Figure 1). Additionally, another study has demonstrated the co-colonization of both resistant and susceptible pneumococcal lineages within the same host [16]. These data suggest that a range of selective pressures, beyond antibiotic use, are influencing the acquisition of distinct antimicrobial resistance genes in both niches. For example, one plausible explanation could be the presence of various micro-niches within the oral cavity, such as dental plaque, which is known to harbor highly intricate and resilient microbial biofilm structures. Conversely, host immune responses in the gut may modulate the colonization and persistence of tetracycline-resistant streptococci, which could potentially impact the prevalence of resistance.

The adaptation of streptococcal species to the intestinal niche appears to be a common phenomenon, irrespective of whether the individual is an IBD patient or a healthy individual. For instance, in individuals P01, P03, C06, and C10, there were 521, 490, 754, and 443 unique genes identified in their intestinal *S. parasanguinis* genomes compared to their oral counterparts, respectively (Table 1). Comparable findings were also noted across the various other streptococcal species scrutinized in this study (Table 1). Notably, these genetic alterations were largely attributed to the acquisition of MGEs, which play diverse roles in functional processes such as coenzyme B12 biosynthesis, Type I restriction endonuclease, accessory secretion systems, and phage acquisition (Supplementary File S1), underscoring the significance of horizontal gene transfer in the within-host evolution of commensal streptococcal species. Furthermore, MGEs such as CRISPR-Cas and restriction-modification systems had a significant impact on niche-specific genotype diversities, facilitating streptococcal genome evolution through the integration of exogenous DNA.

These findings are consistent with those of previous research, which has highlighted the role of MGEs in the adaptation of various pathogenic streptococcal species, including *S. pneumoniae* and *S. dysgalactiae*, to their hosts [6,16,17]. Moreover, these studies have demonstrated the significance of phage-mediated virulence factors, including superantigens and mitogenic factors, in host adaptation. This evidence suggests that horizontal genetic transfer may contribute to the adaptation of streptococcal species to specific niches and the development of host specificity [6,16,17].

It is noteworthy that while phages are typically thought to target specific bacterial species or even specific genetic lineages within a single species, our study yielded intriguing results. We observed distinct clustering of oral and intestinal phages originating from different streptococcal species, exemplified by instances such as C02, C11, P15, and P16 (Figure 2). This mirrors previous findings where streptococcal phages, despite originating from distinct bacterial species, shared the same phylogenetic cluster [18]. Furthermore, this suggests that streptococcal prophages may be undergoing unique evolutionary paths different from their host bacteria, potentially indicating that factors beyond evolutionary relatedness, such as ecological interactions, might play a dominant role in shaping their evolution. However, it is evident from our data that not all individuals host diverse lineages associated with specific signatures. For instance, individual P16 harbored pairs of oral and intestinal genomes of *S. parasanguinis* and *S. infantis2*, respectively, exhibiting remarkable similarity and minimal acquisition of unique genes (Table 1). These findings imply that within certain hosts, streptococcal strains may encounter distinct mutagens that independently drive adaptation to each niche. Nonetheless, it is possible that we currently lack a sufficient number of genomes per niche for certain hosts to enable discrimination via particular genetic signatures.

Remarkably, comparable occurrences of within-host evolution have been documented in various bacterial species. For instance, strains of *Clostridium innocuum* isolated from distinct locations within the intestines of Crohn’s disease patients exhibited notable genetic disparities [19]. Additionally, *Enterococcus gallinarum* demonstrated divergence into distinct lineages specialized for inhabiting either luminal or mucosal niches within the gastrointestinal tract. Mucosally adapted strains of *E. gallinarum*, when compared to ancestral and luminal variants, demonstrated the capability to evade immune surveillance and clearance, thereby instigating heightened inflammation in both intestinal and hepatic tissues. These alterations in bacterial virulence were correlated with non-synonymous mutations or insertion-deletions in specific regulatory genes of *E. gallinarum*, leading to modifications in microbial gene expression profiles and restructuring of cell wall architecture [20]. Similarly, *Lactobacillus* (new nomenclature *Limosilactobacillus*) *reuteri* displayed comparable patterns of divergent evolution and augmented immune evasion in a model of within-host evolution based on mono-colonization. Collectively, these investigations have elucidated within-host evolution as a pivotal modulator of commensal pathogenicity, introducing a distinctive element of unpredictability into the initiation and progression of microbiota-associated diseases [20].

Upon analyzing the genetic diversity at the core genome SNPs level, we found notable variation in the mean PWSD among oral and intestinal strains of the same species within individual subjects across multiple instances. While some individuals exhibited a significant PWSD between oral and intestinal streptococcal genomes, others showed minimal PWSD. Furthermore, our investigation highlights the substantial involvement of within-host streptococcal recombination as a crucial mechanism driving the emergence of distinct intestinal strain variants from their oral progenitors during colonization. These findings suggest an oral cavity origin for these species, followed by transmission to the intestine, where subsequent adaptation to the intestinal niche occurs. Additionally, lineage-specific rates of recombination indicate structured genetic exchange within the streptococcal population, possibly leading to the co-circulation of multiple lineages of the same strain within hosts [21]. This observation implies varying responses to distinct niche selection pressures among lineages within the population, emphasizing the heterogeneous capacities for adaptation across different streptococcal populations. Our findings also unveil distinct niche-specific mutation signatures within intestinal genomes, likely influenced by exposure to host-associated mutagens, such as reactive oxygen species (ROS) generated by immune cells, which are known to induce DNA damage and subsequent mutations. Alternatively, DNA repair processes could be different between oral and intestinal environments [22]. Our findings indicate that the oral and intestinal niches may influence the mutational spectra of streptococcal species, with notable effects observed in *S. infantis1* and *S. infantis2* (Figure 5F). These observations are supported by prior investigations that identified comparable intestinal streptococcal SBS mutational signatures to those found in *E. coli*, highlighting the contribution of additional non-phylogenetic factors in shaping mutational spectra [22]. We suggest that niche-specific mutagens may exert their effects across diverse streptococcal species inhabiting similar environments, such as the intestine.

It is important to consider the potential limitations of this study. The primary limitation of this study is the relatively small cohort size, which restricts the generalizability of the findings. This limitation was largely due to the challenges posed by the SARS-CoV-2 pandemic, which presented significant obstacles to participant recruitment. Furthermore, several factors known to influence the microbiome, including diet, smoking, oral inflammation, and oral hygiene, were not controlled for before sample collection, despite their potential relevance. For example, diets rich in fiber have been associated with an increased abundance of short-chain fatty acid-producing bacteria, while high-fat or high-sugar diets may promote pro-inflammatory microbiota profiles [23]. In light of these limitations, no conclusions can be drawn regarding the relationship between these factors and the microbiome of the study participants. Another noteworthy limitation is the methodology employed, which combined culture-based techniques with short-read WGS to investigate the within-host evolution and adaptation of streptococci to oral and intestinal niches. While culture-based methods provide valuable insights into specific bacterial strains, they inherently limit the ability to capture the full genetic diversity and dynamics within a microbial population. This bias towards cultivable species may result in the underrepresentation of non-culturable or slow-growing bacteria, thereby limiting the understanding of the microbial community and its evolutionary processes. Similarly, short-read WGS, while offering previously unattainable insights into the genetic changes driving streptococcal evolution, has limitations in detecting the full spectrum of MGEs, such as plasmids and insertion sequences. Given the pivotal role that MGEs play in horizontal gene transfer and microbial adaptation, an underrepresentation of these elements could hinder a comprehensive understanding of streptococcal evolution. The restricted sample size also impacts the statistical capacity to ascertain the mutational spectrum of specific streptococcal species, thereby limiting the broader applicability of these findings.

To address these challenges, future studies should integrate complementary sequencing approaches, such as long-read or hybrid sequencing strategies, to enhance the resolution and capture of genetic diversity, including MGEs. Furthermore, longitudinal studies with larger cohorts are essential to improve the robustness and reliability of findings in this field. It is recommended that future studies should also include the administration of standardized questionnaires to obtain detailed information on diet, medications, and lifestyle factors. This will assist in elucidating the intricate interactions that shape the microbiome. Additionally, the use of advanced computational methodologies, such as artificial intelligence algorithms, could facilitate the prediction of evolutionary and adaptive patterns of bacterial strains across different niches.

In conclusion, despite these limitations, our study highlights the complex and dynamic nature of within-host evolution in streptococci, driven by genetic mutations, horizontal gene transfer, and intricate host–pathogen interactions. Understanding niche-specific adaptation mechanisms allows for precise identification of resistance emergence hotspots. This can guide the development of strategies to counteract AMR, including the refinement of antibiotic stewardship programs and the design of niche-specific antimicrobials. By elucidating the intricacies of within-host evolution, particularly the streptococcal adaptation to diverse oral and intestinal niches, we can facilitate the development of innovative approaches to infection management. For example, recognizing the oral cavity as a reservoir opens avenues for microbiome-targeted interventions. Engineering microbial ecosystems could disrupt pathogenic streptococcal populations while bolstering beneficial commensals, reducing infection risk, and limiting the potential for resistance development. Furthermore, insights into the mechanisms of host–pathogen interactions and genetic variability inform vaccine design. Targeting conserved elements crucial for streptococcal survival across oral and intestinal niches may yield vaccines with broader protective efficacy. Ultimately, these efforts may alleviate the global burden of streptococcal infections on public health. Therefore, further investigations are warranted to deepen our comprehension of these mechanisms.

## 4. Materials and Methods

### 4.1. Bacterial Isolates

We included a total of 218 streptococcal isolates (Supplementary Table S1) obtained from saliva and fecal samples collected from 14 IBD patients and 12 age- and gender-matched healthy control subjects between March 2019 to April 2021. Recruitment was limited by the SARS-CoV-2 pandemic, which affected the number of patients and healthy individuals enrolled. Clinical data, including gender, age, disease type, medications, history of bowel surgery, and exclusion criteria, were documented as described in our previous study [3]. Briefly, saliva samples were collected in the morning after participants were instructed to chew paraffin gum to stimulate saliva production. No dietary restrictions were imposed; however, all participants were kindly asked to refrain from eating, drinking, or brushing their teeth for at least 30 min before sample collection. For fecal sampling, a feces catcher (Trafalgar Scientific Ltd., Leicester, UK) and sterile fecal tubes with screw caps (dimensions: 107 × 25 mm, transparent; Sarstedt AG & Co. KG, Nümbrecht, Germany) equipped with spoons were utilized. Participants were instructed to collect the stool sample contamination-free using the feces catcher and transfer at least one gram of feces into the sterile tube using the provided sterile spoon. Among the investigated streptococcal isolates, 93 and 55 isolates were retrieved from saliva samples of IBD patients and healthy control subjects, respectively. Additionally, 33 isolates were retrieved from fecal samples of IBD patients (11/14), while 37 isolates were obtained from fecal samples of healthy control subjects. 

### 4.2. Cultivation and Species Identification

To facilitate the proliferation of Gram-positive bacteria, particularly streptococci, while impeding the growth of Gram-negative bacteria, samples of saliva and feces were streaked onto Columbia colistin-nalidixic acid (CNA) agar supplemented with 5% (*vol*/*vol*) sheep blood (Becton Dickinson GmbH, Heidelberg, Germany). The agar plates were then incubated at 37 °C in an atmosphere containing 8% CO_2_. Sterile 10 μL inoculation loops were employed to evenly distribute the samples across the agar surface, thereby promoting the growth of individual colonies, as previously described [3]. Typically, an incubation period of 24 h was adequate for optimal growth, although, in some instances, an extended period of 48 h was necessary. Species identification was performed using MALDI-TOF MS (Biotyper, Bruker Daltonik GmbH, Bremen, Germany), following the manufacturer’s instructions.

### 4.3. DNA Extraction and Whole-Genome Sequencing

For DNA extraction, bacterial biomass was collected and suspended in 1 mL of 0.9% sodium chloride (NaCl) solution in 1.5 mL Eppendorf tubes. The tubes were centrifuged at 8000 rpm for 1 min, and the supernatant was discarded. The resulting pellets were treated with a mixture of lysozyme and mutanolysin and incubated at 37 °C for 30 min to disrupt the cell walls. Genomic DNA was then extracted using the QIAamp^®^ DNA Mini Kit (Qiagen GmbH, Hilden, Germany), following the manufacturer’s instructions. Subsequently, the genomic DNA was subjected to whole-genome sequencing (WGS) using the Illumina NovaSeq 6000 platform (Illumina, Inc., San Diego, CA, USA) generating 250 base-paired (bp)-end reads. Demultiplexing of all libraries for each sequencing data were performed using Illumina bcl2fastq software v 2.20 and reads with final length less than 20 bases were discarded.

### 4.4. WGS Data Analysis

Sequence reads FASTQ files were passed through the Bactopia pipeline v.1.6 [24]. Briefly, the sequencing data quality and trimming of sequencing adapter remnants from all reads was performed using trimmomatic v.0.39 [25]. Subsequently, the sequencing reads were assembled de novo into contigs using Spades v.3.14.1 software, and assembly quality was evaluated using QUAST v.5.0.2 and CheckM genome v.1.1.3, which are all implemented into Bactopia pipeline [24,26,27]. The generated contigs were annotated using Prokka pipeline v.1.13.0 [28]. These de novo assembled and annotated genomes were used to analyze the Pan-genome via Roary v.3.11.2, while Phandango v.1.3.0 was used to visualize the results [29,30]. The presence or absence of acquired resistance genes was determined via ABRicate v.0.8.132 pipeline with default settings. The public databases ARG-ANNOT and CARD were used as references for detecting the antimicrobial resistance determinants [31,32].

### 4.5. Phage Content Analysis in Streptococcal Genomes

Putative phage content regions within the assembled genomes were identified using PHASTER (https://phaster.ca/ (accessed on 17 November 2022)) [11,12]. The putative phage sequences from each genome were compiled into multifasta files. To conduct a comprehensive analysis of phage sequences across all oral and intestinal streptococcal genomes, multiple sequence alignments were performed using the whole genome alignment tool integrated into CLC Workbench v.23.0.2 (Qiagen GmbH, Hilden, Germany). Subsequently, *K*-mer-based trees were constructed for the putative phage sequences from each healthy control or IBD patient with oral and intestinal genomes, utilizing CLC Workbench with a *K*-mer length of 15 and the distance measure method of Fractional Common *K*-mer Count.

### 4.6. Core Genome Alignment, Recombination, and Mutational Spectrum Analysis

The genomic sequencing reads from *S. salivarius*, *S. infantis*, *S. parasanguinis*, and *S. australis* were aligned to species-specific type reference genomes ATCC 7073, ATCC 700779, ATCC 15912, and ATCC 700641, respectively, using the Snippy pipeline v.4.2.0 [33]. Core genome alignments and multi-sample VCF files were generated using the Snippy-core function. Pairwise SNP differences were calculated using MEGA software v.11 [34]. Furthermore, to investigate recombination events within a single host, we selected genomes from the same individuals and species, represented by more than three sequenced genomes, found in both oral and intestinal environments. The sequencing reads from the acquired FASTQ files were aligned using the Snippy pipeline against the species-specific oral genome obtained from the same host. Homologous recombination events were assessed using Gubbins v.2.4.1 [35]. The mutational spectra for oral and intestinal genomes of each species were estimated from the VCF files and the species-specific reference genomes using the MutTui v.1.1.10 pipeline (https://github.com/chrisruis/MutTui) [22]. MutTui leverages variable site alignment, phylogenetic tree, and reference genome for each dataset and has been previously employed by Ruis et al. to measure mutational spectra associated with distinct bacterial niches [22]. Comparisons of mutational spectra between niches for each species were conducted using the MutTui compare function.

## Figures and Tables

**Table 1:** Number of unique genes identified among oral–gut axis pairs of streptococcal genomes within the same host.

		Sample	
Species	Host	Saliva	Feces
***S. parasanguinis***	**C03**	361	416
	**C06**	523	754
	**C10**	525	443
	**P01**	551	521
	**P03**	491	490
	**P04**	21	78
	**P05**	362	310
	**P07**	4	6
	**P09**	422	365
	**P15**	85	76
	**P16**	12	5
	**P21**	363	517
***S. australis***	**C08**	5	1
	**C11**	537	572
	**P03**	454	467
	**P12**	546	534
***S. infantis1***	**C10**	802	752
	**C10**	883	746
	**P16**	4	6
***S. infantis2***	**C02**	622	625
	**C03**	11	16
	**P04**	1	0
	**P04**	1	1
	**P04**	5	31
	**P15**	687	762
	**P16**	22	6
	**P18**	346	312
***S. salivarius***	**C02**	521	1064
	**C03**	71	27
	**C07**	3	3
	**C10**	14	13
	**C13**	24	11

## Data Availability

Raw data have been made publicly available at the National Center for Biotechnology Information (NCBI) repository, under the project accession number PRJNA971110.

## Supplementary Materials

The following supporting information can be downloaded at: https://www.mdpi.com/article/10.3390/ijms252413507/s1. **Figure S1**. Illustration of the colonization dynamics of oral and intestinal streptococci in both inflammatory bowel disease (IBD) patients and healthy controls. Panel A displays the prevalence of different streptococcal species detected in oral and intestinal samples from both groups. Panel B highlights significant variations in antimicrobial resistance genes between the oral and intestinal niches. **Figure S2**. Gubbins-based recombinational analysis and phylogenetic tree reconstruction illustrate oral and intestinal streptococcal genomes from the same host, showing genomes with a low frequency of recombination events. Red blocks show recombination events shared among multiple isolates. Blue blocks denote recombination events that are unique to individual isolates. The black bars represent the number of observed recombination events covering specific positions in the genome. **Figure S3**. Single base substitution (SBS) spectra across all four investigated streptococcal species within both oral and intestinal niches. The reconstruction of SBS utilized core genome alignments and a multi-sample VCF file containing genotype tags for all discovered alleles within the oral and intestinal genomes of each species, processed using the MutTui pipeline. The left-side panels delineate the SBS spectra for the oral niche, while the right-side panels illustrate the SBS spectra for the intestinal niche. Panels A–E present SBS spectrum plots, representing the counts of mutations detected for *S. australis*, *S. salivarius*, *S. infantis1*, *S. infantis2*, and *S. parasanguinis*, respectively. **Table S1**. Metadata and assembly statistics of the 218 streptococcal genomes investigated in this study. **Table S2**. Antimicrobial resistance genes that were detected among the 218 streptococcal genomes. **Table S3**. Estimates of evolutionary divergence between sequences. **Supplementary File S1**. The within-host comparative analysis of oral and intestinal streptococcal genomes across all species was conducted using the BLAST Ring Image Alignment (BRIG) tool. This analysis included genomes obtained from both inflammatory bowel disease (IBD) patients and healthy controls, focusing on those with at least one oral-gut pair or multiple genomes originating from the same niche. The streptococcal species analyzed were *Streptococcus parasanguinis*, *S. infantis1*, *S. infantis2*, *S. salivarius*, and *S. australis*. Each genome is represented by a colored ring, and whenever possible, gut (fecal) genomes were used as the reference (inner ring) for comparison.
