# Supplementary material for: Insights into Within-Host Evolution and Dynamics of Oral and Intestinal Streptococci Unveil Niche Adaptation
Source: Int J Mol Sci. 2024 Dec 17;25(24):13507. doi: 10.3390/ijms252413507 (PMC11727833; doi:10.3390/ijms252413507)
Supplement: Supplementary file 1 [file ijms-25-13507-s001.zip › Supplementary material20241216/FigureS1.pdf]

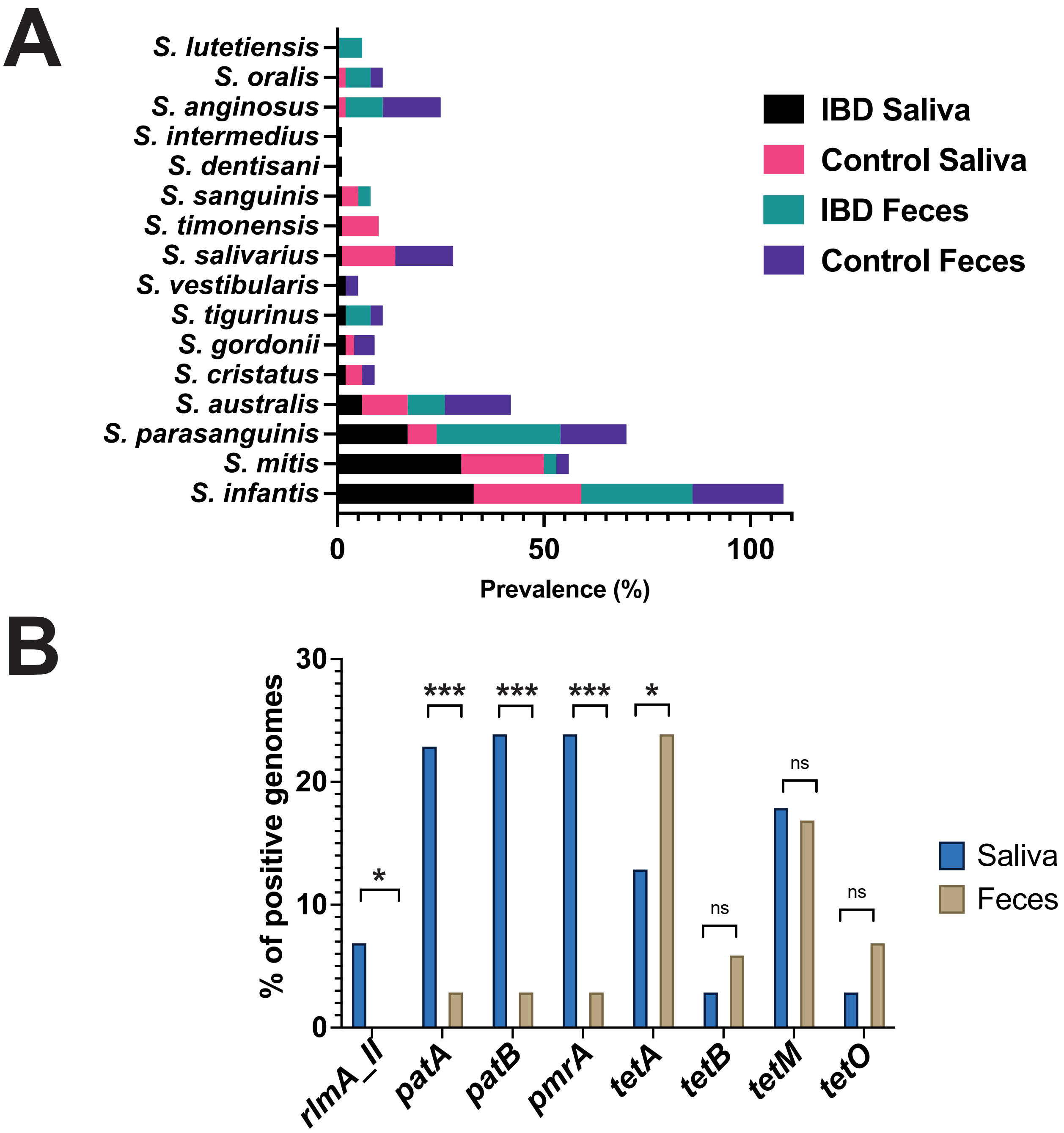

Figure S1. Illustration of the colonization dynamics of oral and intestinal streptococci in both inflammatory bowel disease (IBD) patients and healthy controls. Panel A displays the prevalence of different streptococcal species detected in oral and intestinal samples from both groups. Panel B highlights significant variations in antimicrobial resistance genes between the oral and intestinal niches.
