# Supplementary material for: Insights into Within-Host Evolution and Dynamics of Oral and Intestinal Streptococci Unveil Niche Adaptation
Source: Int J Mol Sci. 2024 Dec 17;25(24):13507. doi: 10.3390/ijms252413507 (PMC11727833; doi:10.3390/ijms252413507)
Supplement: Supplementary file 1 [file ijms-25-13507-s001.zip › Supplementary material20241216/FigureS2.pdf]

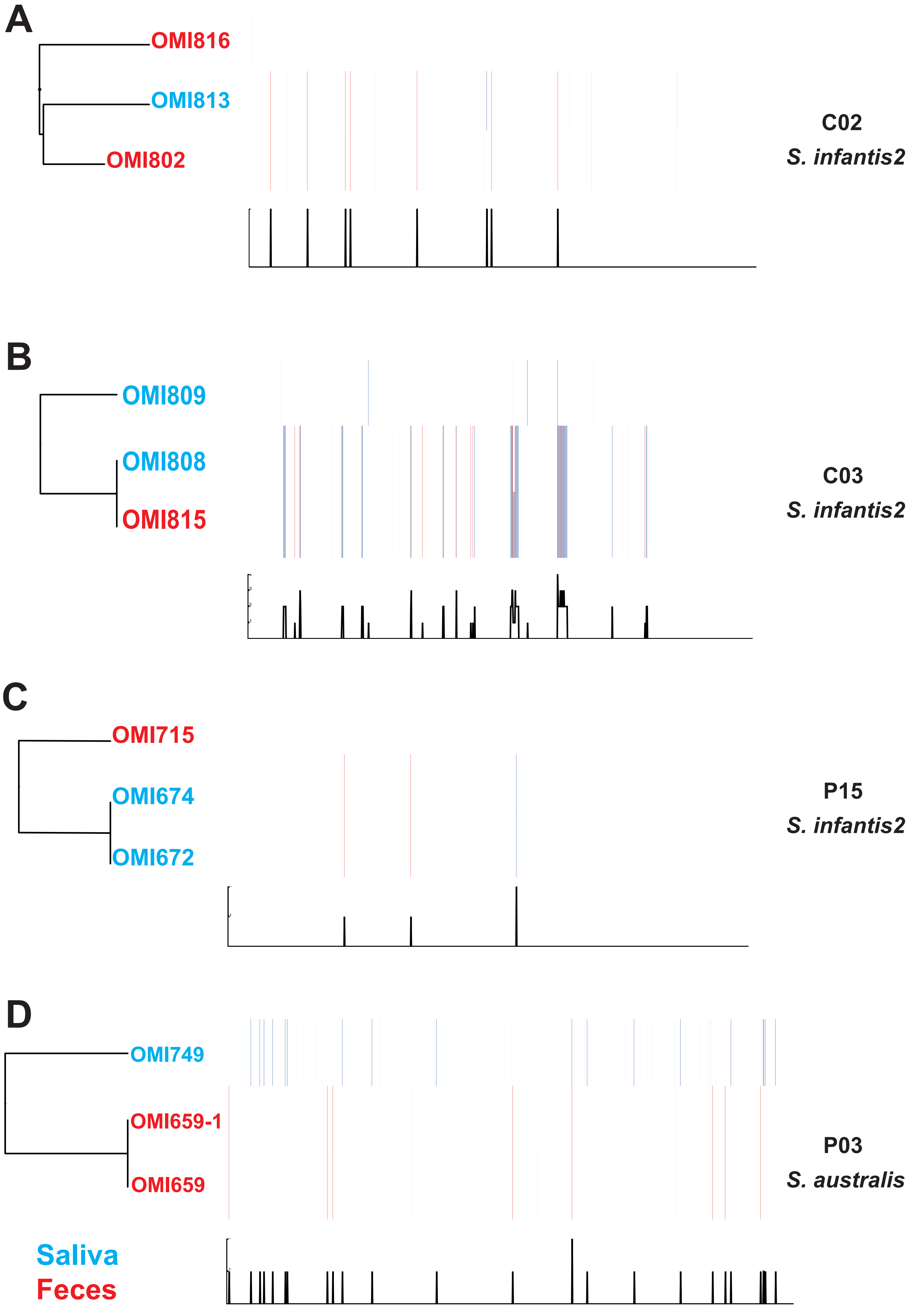

A
OMI816
OMI813
OMI802
C02
S. infantis2
B
OMI809
OMI808
OMI815
C03
S. infantis2
C
OMI715
OMI674
OMI672
P15
S. infantis2
D
OMI749
OMI659-1
OMI659
P03
S. australis
Saliva
Feces

Figure S2. Gubbins-based recombinational analysis and phylogenetic tree reconstruction illustrate oral and intestinal streptococcal genomes from the same host, showing genomes with a low frequency of recombination events. Red blocks show recombination events shared among multiple isolates. Blue blocks denote recombination events that are unique to individual isolates. The black bars represent the number of observed recombination events covering specific positions in the genome.
