# Supplementary material for: Insights into Within-Host Evolution and Dynamics of Oral and Intestinal Streptococci Unveil Niche Adaptation
Source: Int J Mol Sci. 2024 Dec 17;25(24):13507. doi: 10.3390/ijms252413507 (PMC11727833; doi:10.3390/ijms252413507)
Supplement: Supplementary file 1 [file ijms-25-13507-s001.zip › Supplementary material20241216/FigureS3.pdf]

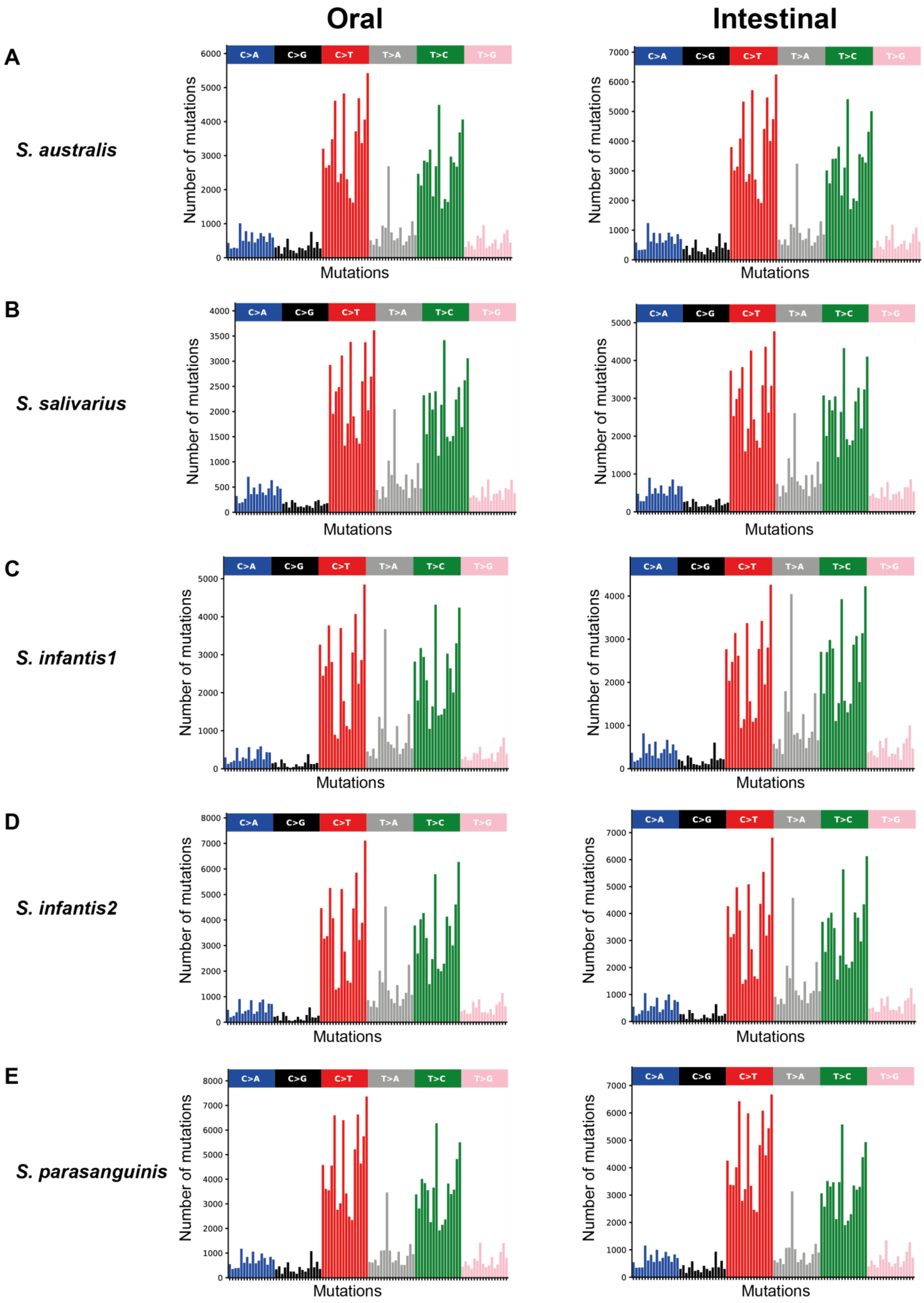

Oral
Intestinal
A
S. australis
B
S. salivarius
C
S. infantis1
D
S. infantis2
E
S. parasanguinis
C>A
C>G
C>T
T>A
T>C
T>G
Number of mutations
Mutations

Figure S3. Single base substitution (SBS) spectra across all four investigated streptococcal species within both oral and intestinal niches. The reconstruction of SBS utilized core genome alignments and a multi-sample VCF file containing genotype tags for all discovered alleles within the oral and intestinal genomes of each species, processed using the MutTui pipeline. The left-side panels delineate the SBS spectra for the oral niche, while the right-side panels illustrate the SBS spectra for the intestinal niche. Panels A-E present SBS spectrum plots, representing the counts of mutations detected for *S. australis*, *S. salivarius*, *S. infantis1*, *S. infantis2*, and *S. parasanguinis*, respectively.
