# Supplementary material for: Insights into Within-Host Evolution and Dynamics of Oral and Intestinal Streptococci Unveil Niche Adaptation
Source: Int J Mol Sci. 2024 Dec 17;25(24):13507. doi: 10.3390/ijms252413507 (PMC11727833; doi:10.3390/ijms252413507)
Supplement: Supplementary file 1 [file ijms-25-13507-s001.zip › Supplementary material20241216/Supplementary File S1.pdf]

Supplementary File S1. The within-host comparative analysis of oral and intestinal streptococcal genomes across all species was conducted using the BLAST Ring Image Alignment (BRIG) tool. This analysis included genomes obtained from both inflammatory bowel disease (IBD) patients and healthy controls, focusing on those with at least one oral-gut pair or multiple genomes originating from the same niche. The streptococcal species analyzed were *Streptococcus parasanguinis*, *S. infantis1*, *S. infantis2*, *S. salivarius,* and *S. australis.* Each genome is represented by a colored ring, and whenever possible, gut (fecal) genomes were used as the reference (inner ring) for comparison.

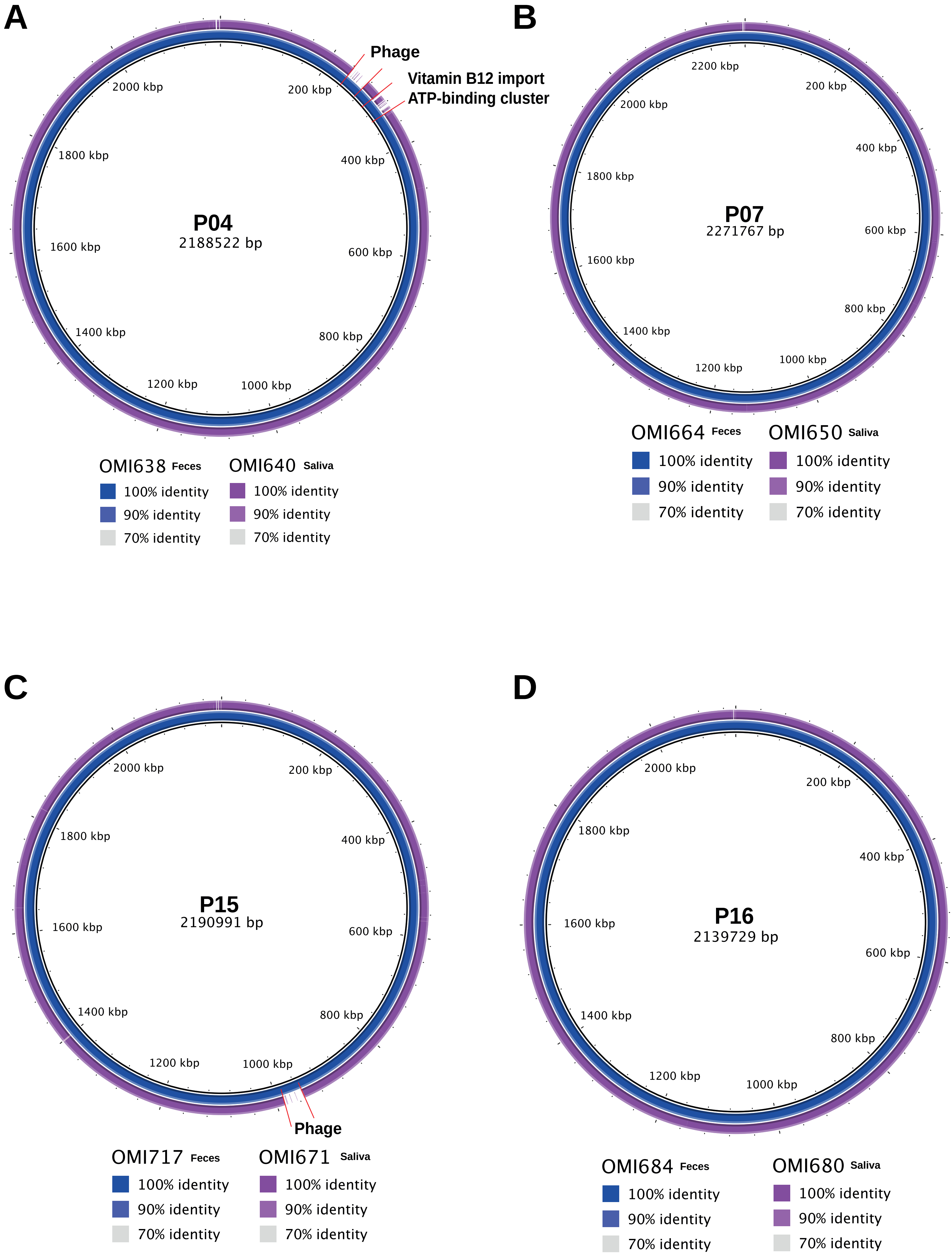

S. parasanguinis

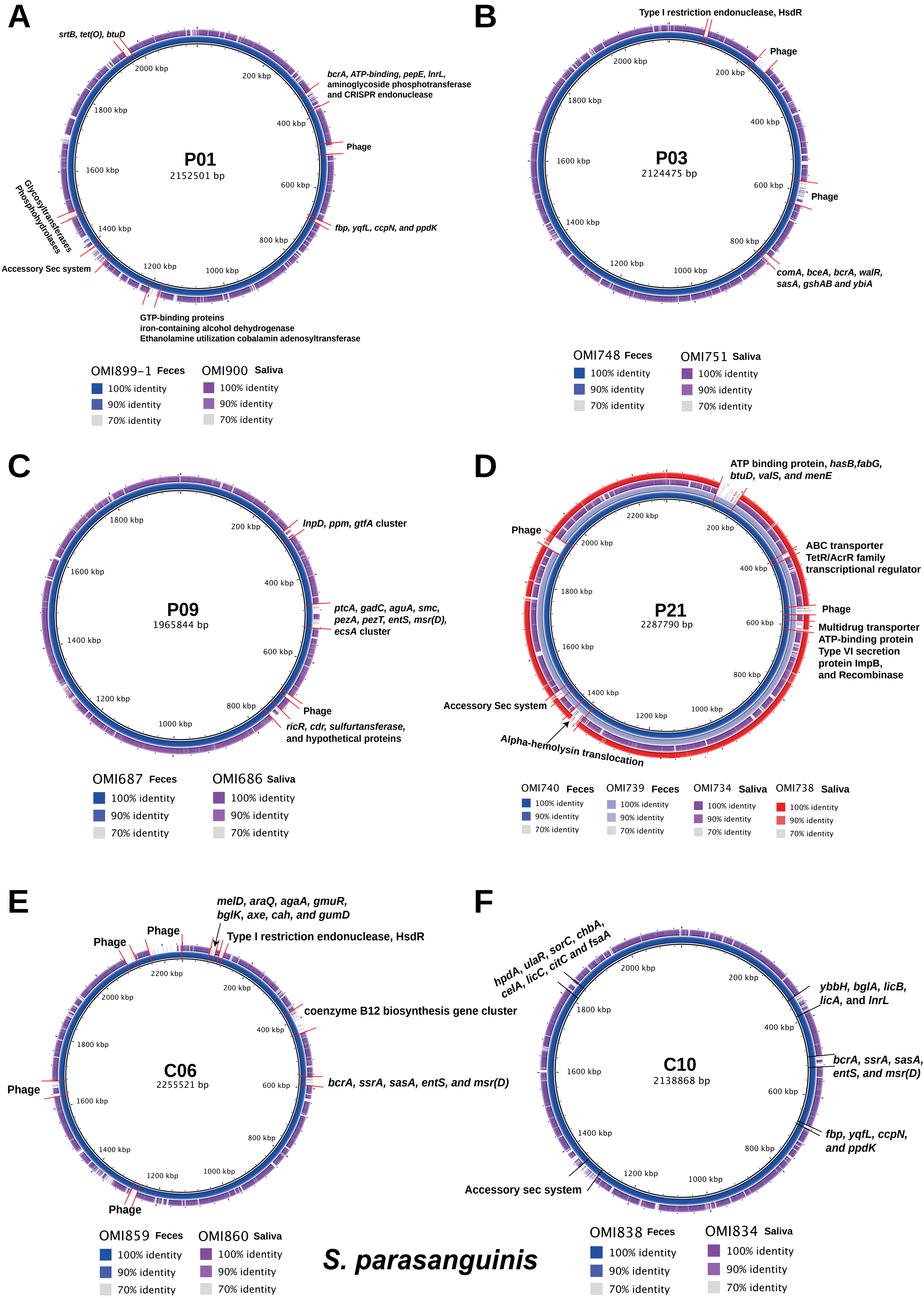
A
srtB, tet(O), btuD
bcrA, ATP-binding, pepE, lnrL, aminoglycoside phosphotransferase and CRISPR endonuclease
Phage
P01
2152501 bp
fbp, yqfL, ccpN, and ppdK
Glycosyltransferases
Phosphohydrolases
Accessory Sec system
GTP-binding proteins
iron-containing alcohol dehydrogenase
Ethanolamine utilization cobalamin adenosyltransferase
OMI899-1 Feces
100% identity
90% identity
70% identity
OMI900 Saliva
100% identity
90% identity
70% identity
B
Type I restriction endonuclease, HsdR
Phage
P03
2124475 bp
Phage
comA, bceA, bcrA, walR, sasA, gshAB and ybiA
OMI748 Feces
100% identity
90% identity
70% identity
OMI751 Saliva
100% identity
90% identity
70% identity
C
lnpD, ppm, gtfA cluster
P09
1965844 bp
ptcA, gadC, aguA, smc, pezA, pezT, entS, msr(D), ecsA cluster
Phage
ricR, cdr, sulfurtansferase, and hypothetical proteins
OMI687 Feces
100% identity
90% identity
70% identity
OMI686 Saliva
100% identity
90% identity
70% identity
D
ATP binding protein, hasB, fabG, btuD, valS, and menE
Phage
ABC transporter
TetR/AcrR family transcriptional regulator
P21
2287790 bp
Phage
Multidrug transporter
ATP-binding protein
Type VI secretion protein ImpB, and Recombinase
Accessory Sec system
Alpha-hemolysin translocation
OMI740 Feces
100% identity
90% identity
70% identity
OMI739 Feces
100% identity
90% identity
70% identity
OMI734 Saliva
100% identity
90% identity
70% identity
OMI738 Saliva
100% identity
90% identity
70% identity
E
melD, araQ, agaA, gmuR, bglK, axe, cah, and gumD
Phage
Phage
Type I restriction endonuclease, HsdR
coenzyme B12 biosynthesis gene cluster
C06
2255521 bp
Phage
bcrA, ssrA, sasA, entS, and msr(D)
Phage
OMI859 Feces
100% identity
90% identity
70% identity
OMI860 Saliva
100% identity
90% identity
70% identity
F
hpdA, ulaR, sorC, chbA, celA, licC, citC and fsaA
ybbH, bglA, licB, licA, and lnrL
C10
2138868 bp
bcrA, ssrA, sasA, entS, and msr(D)
fbp, yqfL, ccpN, and ppdK
Accessory sec system
OMI838 Feces
100% identity
90% identity
70% identity
OMI834 Saliva
100% identity
90% identity
70% identity
S. parasanguinis

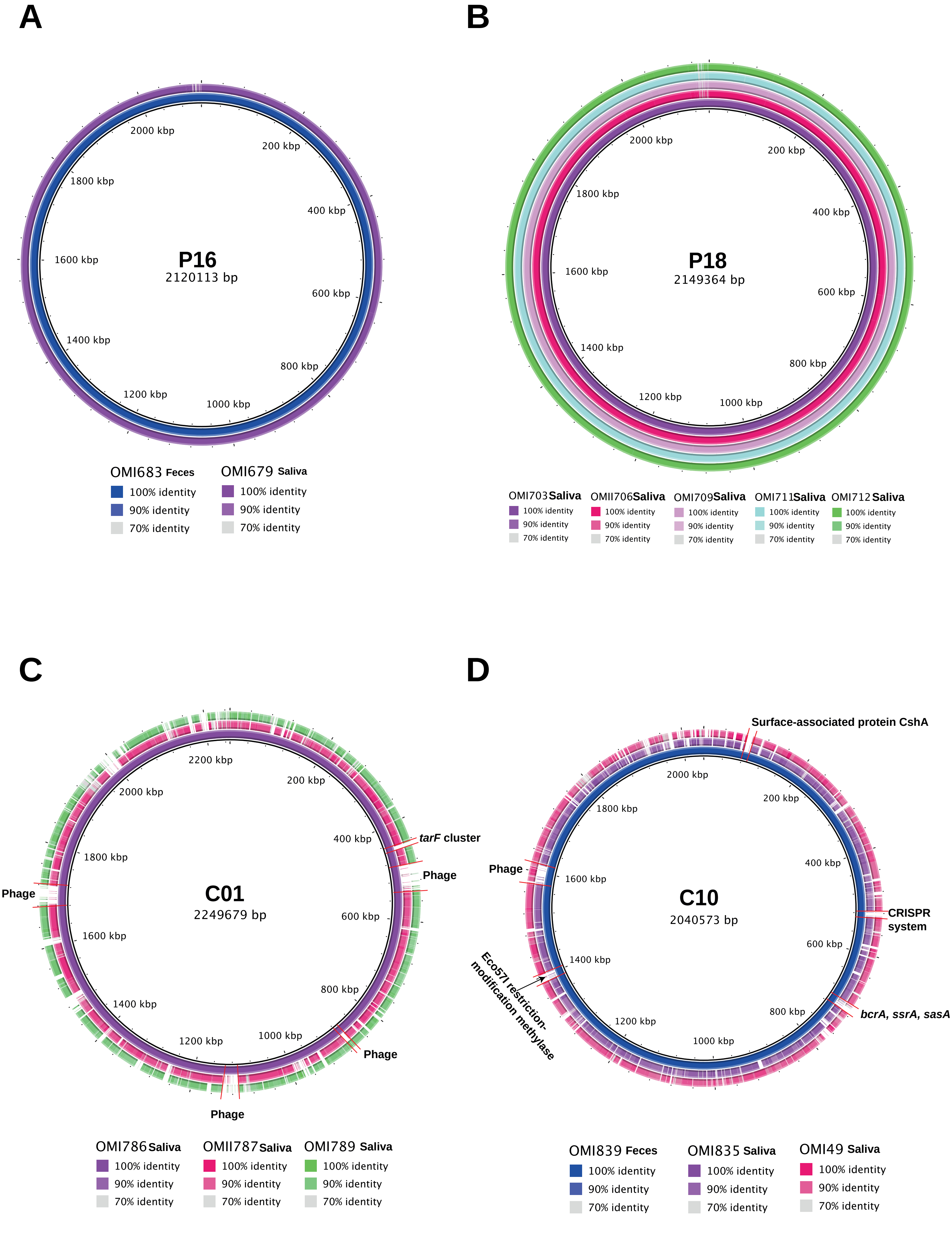
A
2000 kbp
200 kbp
1800 kbp
400 kbp
1600 kbp
P16
2120113 bp
600 kbp
1400 kbp
800 kbp
1200 kbp
1000 kbp
OMI683 Feces
100% identity
90% identity
70% identity
OMI679 Saliva
100% identity
90% identity
70% identity
B
2000 kbp
200 kbp
1800 kbp
400 kbp
1600 kbp
P18
2149364 bp
600 kbp
1400 kbp
800 kbp
1200 kbp
1000 kbp
OMI703Saliva
100% identity
90% identity
70% identity
OMII706Saliva
100% identity
90% identity
70% identity
OMI709Saliva
100% identity
90% identity
70% identity
OMI711Saliva
100% identity
90% identity
70% identity
OMI712Saliva
100% identity
90% identity
70% identity
C
2200 kbp
200 kbp
2000 kbp
400 kbp
tarF cluster
1800 kbp
Phage
Phage
C01
2249679 bp
600 kbp
1600 kbp
800 kbp
1400 kbp
1200 kbp
1000 kbp
Phage
Phage
OMI786Saliva
100% identity
90% identity
70% identity
OMII787Saliva
100% identity
90% identity
70% identity
OMI789 Saliva
100% identity
90% identity
70% identity
D
Surface-associated protein CshA
2000 kbp
1800 kbp
200 kbp
Phage
1600 kbp
400 kbp
C10
2040573 bp
CRISPR
system
600 kbp
1400 kbp
Eco57I restriction-
modification methylase
800 kbp
bcrA, ssrA, sasA
1200 kbp
1000 kbp
OMI839 Feces
100% identity
90% identity
70% identity
OMI835 Saliva
100% identity
90% identity
70% identity
OMI49 Saliva
100% identity
90% identity
70% identity

S. infantis1

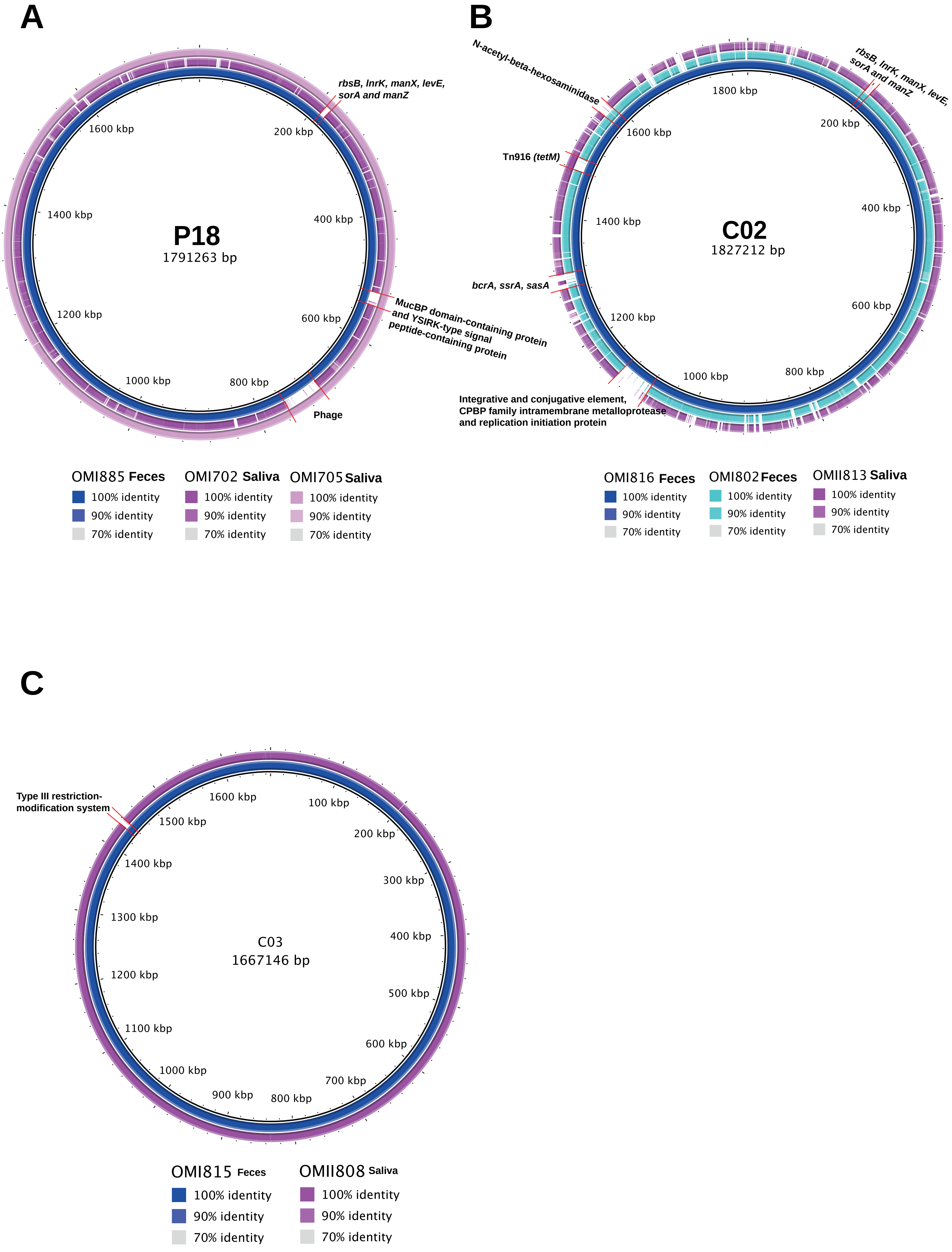
A
P18
1791263 bp
200 kbp
400 kbp
600 kbp
800 kbp
1000 kbp
1200 kbp
1400 kbp
1600 kbp
rbsB, lnrK, manX, levE, sorA and manZ
MucBP domain-containing protein and YSIRK-type signal peptide-containing protein
Phage
OMI885 Feces
OMI702 Saliva
OMI705 Saliva
100% identity
90% identity
70% identity
B
C02
1827212 bp
200 kbp
400 kbp
600 kbp
800 kbp
1000 kbp
1200 kbp
1400 kbp
1600 kbp
1800 kbp
N-acetyl-beta-hexosaminidase
rbsB, lnrK, manX, levE, sorA and manZ
Tn916 (tetM)
bcrA, ssrA, sasA
Integrative and conjugative element, CPBP family intramembrane metalloprotease and replication initiation protein
OMI816 Feces
OMI802 Feces
OMII813 Saliva
100% identity
90% identity
70% identity
C
C03
1667146 bp
100 kbp
200 kbp
300 kbp
400 kbp
500 kbp
600 kbp
700 kbp
800 kbp
900 kbp
1000 kbp
1100 kbp
1200 kbp
1300 kbp
1400 kbp
1500 kbp
1600 kbp
Type III restriction-modification system
OMI815 Feces
OMII808 Saliva
100% identity
90% identity
70% identity

S. infantis2

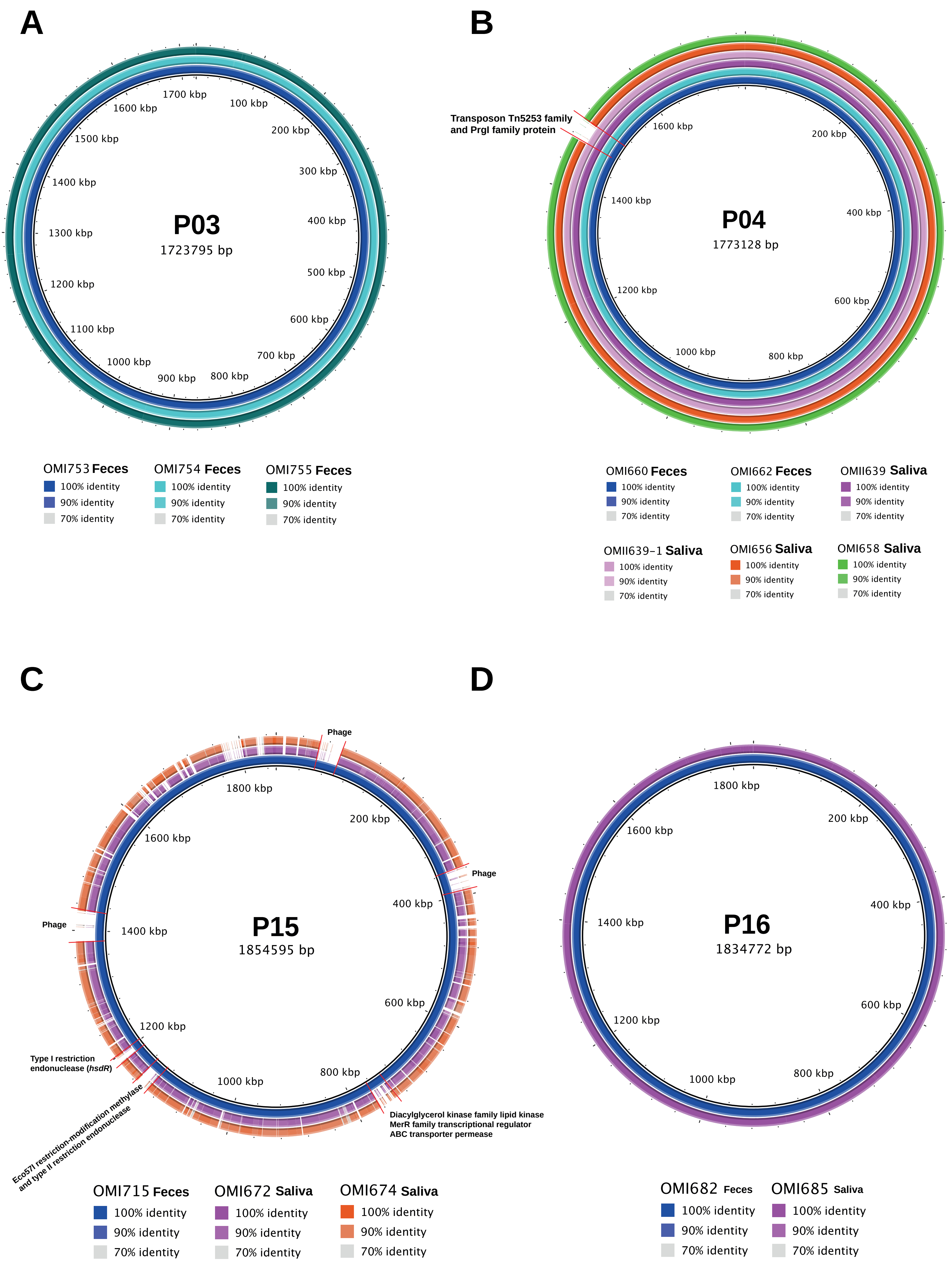
A
P03
1723795 bp
100 kbp
200 kbp
300 kbp
400 kbp
500 kbp
600 kbp
700 kbp
800 kbp
900 kbp
1000 kbp
1100 kbp
1200 kbp
1300 kbp
1400 kbp
1500 kbp
1600 kbp
1700 kbp
OMI753 Feces
100% identity
90% identity
70% identity
OMI754 Feces
100% identity
90% identity
70% identity
OMI755 Feces
100% identity
90% identity
70% identity
B
P04
1773128 bp
Transposon Tn5253 family
and PrgI family protein
200 kbp
400 kbp
600 kbp
800 kbp
1000 kbp
1200 kbp
1400 kbp
1600 kbp
OMI660 Feces
100% identity
90% identity
70% identity
OMI662 Feces
100% identity
90% identity
70% identity
OMI639 Saliva
100% identity
90% identity
70% identity
OMI639-1 Saliva
100% identity
90% identity
70% identity
OMI656 Saliva
100% identity
90% identity
70% identity
OMI658 Saliva
100% identity
90% identity
70% identity
C
P15
1854595 bp
Phage
Phage
Phage
200 kbp
400 kbp
600 kbp
800 kbp
1000 kbp
1200 kbp
1400 kbp
1600 kbp
1800 kbp
Type I restriction
endonuclease (hsdR)
Eco57I restriction-modification methylase
and type II restriction endonuclease
Diacylglycerol kinase family lipid kinase
MerR family transcriptional regulator
ABC transporter permease
OMI715 Feces
100% identity
90% identity
70% identity
OMI672 Saliva
100% identity
90% identity
70% identity
OMI674 Saliva
100% identity
90% identity
70% identity
D
P16
1834772 bp
200 kbp
400 kbp
600 kbp
800 kbp
1000 kbp
1200 kbp
1400 kbp
1600 kbp
1800 kbp
OMI682 Feces
100% identity
90% identity
70% identity
OMI685 Saliva
100% identity
90% identity
70% identity

S. infantis2

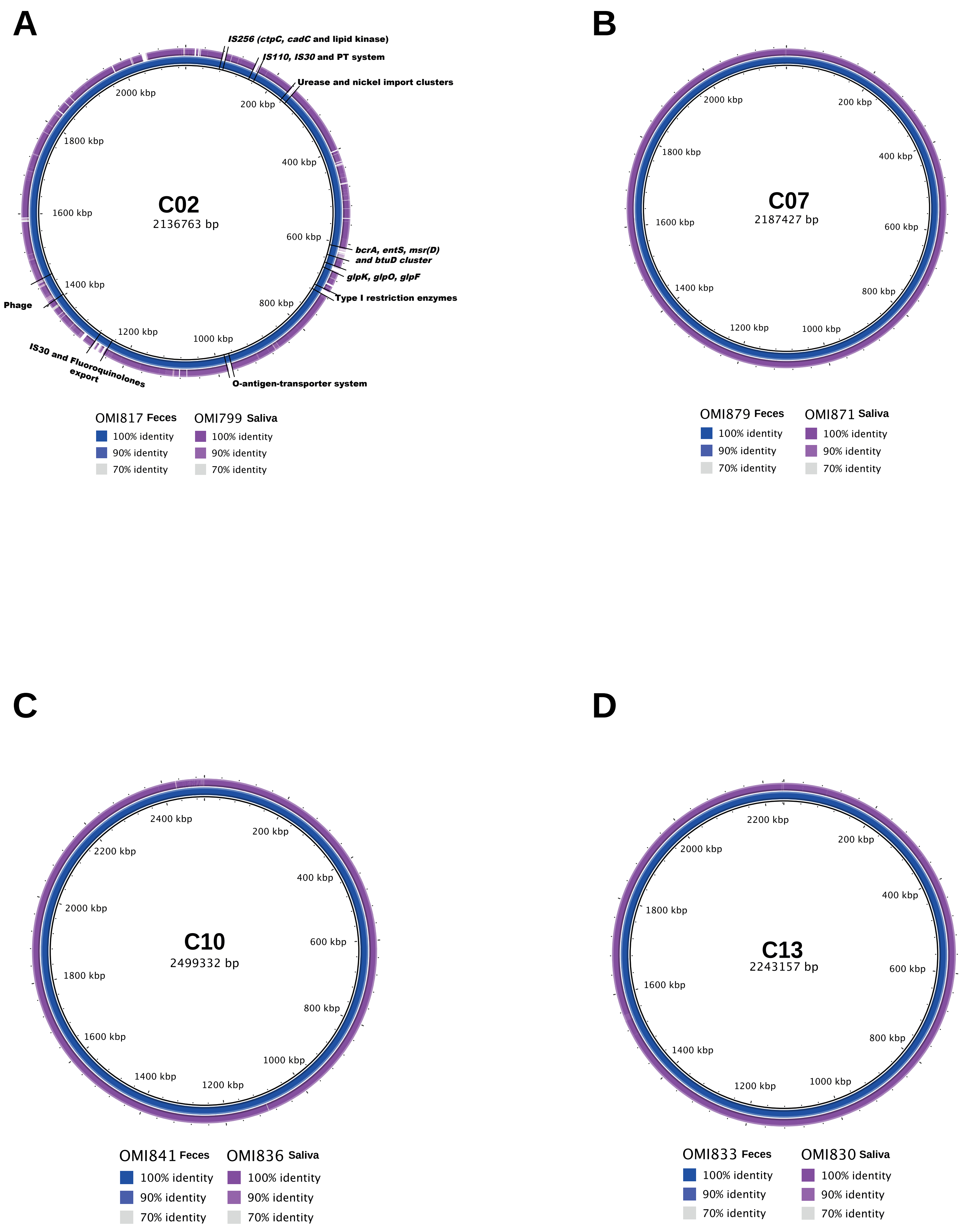

S. salivarius

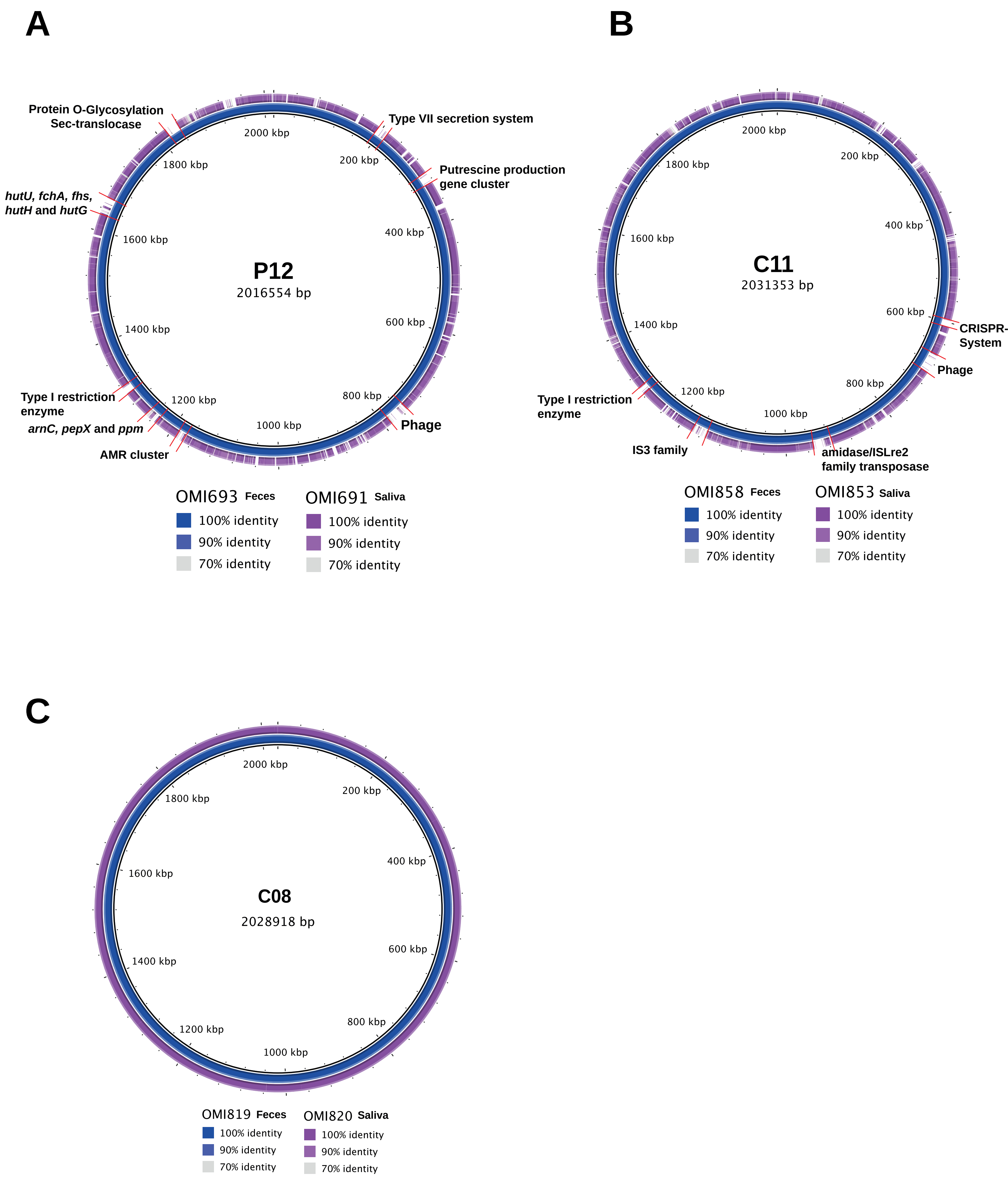
A
Protein O-Glycosylation
Sec-translocase
Type VII secretion system
Putrescine production
gene cluster
hutU, fchA, fhs,
hutH and hutG
P12
2016554 bp
2000 kbp
200 kbp
400 kbp
600 kbp
800 kbp
1000 kbp
1200 kbp
1400 kbp
1600 kbp
1800 kbp
Type I restriction
enzyme
arnC, pepX and ppm
AMR cluster
Phage
OMI693 Feces
100% identity
90% identity
70% identity
OMI691 Saliva
100% identity
90% identity
70% identity
B
C11
2031353 bp
2000 kbp
200 kbp
400 kbp
600 kbp
800 kbp
1000 kbp
1200 kbp
1400 kbp
1600 kbp
1800 kbp
CRISPR-
System
Phage
Type I restriction
enzyme
IS3 family
amidase/ISLre2
family transposase
OMI858 Feces
100% identity
90% identity
70% identity
OMI853 Saliva
100% identity
90% identity
70% identity
C
C08
2028918 bp
2000 kbp
200 kbp
400 kbp
600 kbp
800 kbp
1000 kbp
1200 kbp
1400 kbp
1600 kbp
1800 kbp
OMI819 Feces
100% identity
90% identity
70% identity
OMI820 Saliva
100% identity
90% identity
70% identity

S. australis
